# Connectivity-Based Topographical Changes of the Corpus Callosum During Aging

**DOI:** 10.3389/fnagi.2021.753236

**Published:** 2021-10-20

**Authors:** Yuchen Liu, Chih-Chin Heather Hsu, Chu-Chung Huang, Yajuan Zhang, Jiajia Zhao, Shih-Jen Tsai, Liang-Kung Chen, Ching-Po Lin, Chun-Yi Zac Lo

**Affiliations:** ^1^Institute of Science and Technology for Brain-Inspired Intelligence, Fudan University, Shanghai, China; ^2^Institute of Neuroscience, National Yang Ming Chiao Tung University, Taipei, Taiwan; ^3^Center of Geriatrics and Gerontology, Taipei Veterans General Hospital, Taipei, Taiwan; ^4^Shanghai Key Laboratory of Brain Functional Genomics (Ministry of Education), Affiliated Mental Health Center (ECNU), School of Psychology and Cognitive Science, East China Normal University, Shanghai, China; ^5^Shanghai Changning Mental Health Center, Shanghai, China; ^6^Department of Psychiatry, Taipei Veterans General Hospital, Taipei, Taiwan; ^7^Division of Psychiatry, Faculty of Medicine, National Yang Ming Chiao Tung University, Taipei, Taiwan; ^8^Institute of Brain Science, National Yang Ming Chiao Tung University, Taipei, Taiwan; ^9^Aging and Health Research Center, National Yang Ming Chiao Tung University, Taipei, Taiwan; ^10^Taipei Municipal Gan-Dau Hospital, Taipei, Taiwan

**Keywords:** diffusion MRI, tractography, functional networks, segmentation, atlas, aging trajectory

## Abstract

**Background:** The corpus callosum (CC) is the most prominent white matter connection for interhemispheric information transfer. It is implicated in a variety of cognitive functions, which tend to decline with age. The region-specific projections of the fiber bundles with microstructural heterogeneity of the CC are associated with cognitive functions and diseases. However, how the CC is associated with the information transfer within functional networks and the connectivity changes during aging remain unclear. Studying the CC topography helps to understand the functional specialization and age-related changes of CC subregions.

**Methods:** Diffusion tractography was used to subdivide the CC into seven subregions from 1,086 healthy volunteers within a wide age range (21–90 years), based on the connections to the cortical parcellations of the functional networks. Quantitative diffusion indices and connection probability were calculated to study the microstructure differences and age-related changes in the CC subregions.

**Results:** According to the population-based probabilistic topography of the CC, part of the default mode network (DMN) and limbic network (LN) projected fibers through the genu and rostrum; the frontoparietal network (FPN), ventral attention network (VA) and somatomotor networks (SM) were interconnected by the CC body; callosal fibers arising from the part of the default mode network (DMN), dorsal attention network (DA) and visual network (VIS) passed through the splenium. Anterior CC subregions interconnecting DMN, LN, FPN, VA, and SM showed lower fractional anisotropy (FA) and higher mean diffusivity (MD) and radial diffusivity (RD) than posterior CC subregions interconnecting DA and VIS. All the CC subregions showed slightly increasing FA and decreasing MD, RD, and axial diffusivity (AD) at younger ages and opposite trends at older ages. Besides, the anterior CC subregions exhibited larger microstructural and connectivity changes compared with the posterior CC subregions during aging.

**Conclusion:** This study revealed the callosal subregions related to functional networks and uncovered an overall “anterior-to-posterior” region-specific changing trend during aging, which provides a baseline to identify the presence and timing of callosal connection states.

## Introduction

The corpus callosum (CC) represents the most extensive commissural pathway that connects the cortical regions of the contralateral hemispheres, achieving interhemispheric integration and transfer of information. The structure of the CC contains different axonal diameters and densities, which vary by region. For example, the fiber diameters in the anterior CC (genu) are generally small, whereas those in the posterior midbody are larger (Lamantia and Rakic, 1990; Aboitiz et al., 1992a). The connections of the callosal fibers also exhibit differences, such that small-diameter fibers primarily project to higher-order processing cortical areas, while large diameter fibers tend to connect the visual and somatosensory cortices (Innocenti, 1986; Lamantia and Rakic, 1990; Aboitiz et al., 1992b; Innocenti et al., 1995). This heterogeneity of fiber composition and connections in the CC provides the structural foundation for the differentiation of interhemispheric transfer capacity and velocity between various cortical areas (Aboitiz et al., 1992a, 2003). Studying the CC topography helps to understand the functional specialization of the CC subregions and makes it possible to estimate the cognitive/behavioral damages for the pre-neurosurgical planning of corpus callosotomy (Chao et al., 2009; Vnva et al., 2020).

The structure of the CC is subdivided by vertical lines spaced at fractions of the maximal anterior-posterior length or equal angular rays from the callosal centroid, which are widely used in clinical practice (Clarke et al., 1989; Witelson, 1989; Duara et al., 1991). However, these partitioning methods are based on geometry but not the neurological composition or white matter connections. Advanced diffusion magnetic resonance imaging (dMRI) provides an approach to probe the microstructural information of white matter and estimate the structural connectivity in the human brain. Several tractography-based partitioning approaches have been proposed to divide the CC structure based on the reconstructed streamlines that pass through it and connect it to specific cortical terminations (Huang et al., 2005; Park et al., 2008; Chao et al., 2009). For example, Park et al. (2008) used diffusion tensor imaging (DTI) to present CC population connectivity maps according to 47 semi-automatically partitioned cortical subregions. Similarly, another study presented a cortical cytoarchitectural subregions-based parcellation of the CC using HARDI-based tractography (Chao et al., 2009). These studies and others show that tractography-based partitioning methods provide a more rational subdivision of the CC to link the association between the CC segments and cerebral subsystems concerning distinct functions of the human brain.

The CC plays a critical role in cognitive functions, such as processing speed, visuospatial memory and motor coordination (van der Knaap and van der Ham, 2011; Frederiksen, 2013). DTI studies have shown that the white matter microstructure of the CC is related to cortical activation, reading ability and intelligence (Dougherty et al., 2007; Putnam et al., 2008; Kontis et al., 2009). The work of Dougherty et al. (2005) presented the occipital-callosal projections that pass through the splenium of the CC and project to a functionally defined region-of-interest (ROI). In a functional MRI study of the patients who underwent complete corpus callosotomy, a remarkable reduction of the interhemispheric functional connectivity has been shown among all resting state networks (RSNs), but the subjects who underwent partial corpus callosotomy and retained splenium fibers showed a slight decline in the interhemispheric functional connectivity of the visual network; these results confirm that the splenium fiber bundle is involved in the interhemispheric visual information transfer (Roland et al., 2017). This evidence indicates that the functional integrity of interhemispheric processing is affected by the structural changes in the CC. Hence, linking the callosal projections to functionally organized networks can reveal the functional specialization of the callosal segments.

Morphometric analysis in the elderly has found that the size of the anterior part of the CC decreased (Weis et al., 1993). Similarly, an anteroposterior gradient of an age-related decline in the CC has been revealed through DTI studies. Lower fractional anisotropy (FA) and higher mean diffusion (MD) have been found in anterior callosal fibers compared with posterior ones (genu vs. splenium), and more prominent age-related declines have been revealed in the anterior sections compared with the posterior ones (Sullivan and Pfefferbaum, 2006; Sullivan et al., 2010). Lebel et al. (2010) found an overall “outer-to-inner” trend that MD rising and FA dropping begin earlier and more rapidly in the anterior and posterior callosal segments than the central area. These region-specific changes in the CC may affect the transfer of information between the cortical areas interconnected by the CC (Sullivan et al., 2010). However, the association of the CC with the information transfer within functional networks and its subregional aging progress remain unclear. Studying the changes in the CC subregions linking functionally organized networks can provide information that is directly relevant to the function and essential for determining the role played by the CC in age-related functional and cognitive decline.

In this study, we first presented the tractography-based segmentation of the CC that is related to functionally organized networks. Then, we investigate the age-related microstructural changes and connectivity of each callosal segment. To achieve this, we analyzed the dMRI from a large number of healthy subjects (*n* = 1,086) within a wide age range (21–90 years). Afterward, we used the diffusion tractography-based approach to track the CC fibers that interconnect functionally organized networks, including the visual, sensory-motor functional networks and higher-order association networks (Yeo et al., 2011). Then we parcellated the CC by the neural projections. The participants were divided into seven groups by age to compare the connection probability of each callosal segment among different age ranges. The diffusion indices of the CC segments were established to estimate the aging patterns of each CC segment and compare the microstructural differences among CC segments. We hypothesized that the callosal segments had different compositions and showed region-specific changes along the normal aging process.

## Materials and Methods

### Participants

We collected the MRI data of 1,086 healthy individuals (559 females/527 males) aged 21–90 years. The participants came from two cohorts. The first cohort included 785 community-based aged residents (age range: 50–87) with household registration in the I-Lan country of Taiwan (Liu et al., 2014). The second cohort included 301 participants (age range: 21–89) recruited through advertisements in local communities and universities of northern Taiwan. All the participants were self-reported with good visual and auditory functional abilities, no medical history of significant neurological or psychiatric diseases, and were able to take the cognitive tests. The standard criterion of the intactness of the global cognitive performance for each participant was defined by Mini-Mental State Examination (MMSE) (Folstein et al., 1975) raw scores, where well-educated people (education ≥ 6 years) had a score greater than 24, while less educated people (education < 6 years) showed a score of at least 14 (Sun et al., 2014). The two cohort experiments were separately approved by the Institutional Review Board of Taipei Veterans General Hospital and the Institutional Review Board of National Yang-Ming University. All the participants provided informed consent after being adequately informed of the study.

### Image Acquisition and Preprocessing

MRI scans of the two cohorts were acquired using the same 3T Siemens MR scanner (Siemens MAGNETOM Tim Trio, Erlangen, Germany) with identical imaging protocols, equipped with a 12-channel head coil at National Yang-Ming University, Taipei, Taiwan. T_1_-weighted (T_1_w) images were scanned using the magnetization-prepared rapid gradient echo (MPRAGE) protocol with the following imaging parameters: TR/TE/TI = 3,500/3.5/1,100 ms, flip angle = 7°, voxel size = 1 mm × 1 mm × 1 mm without interslice gap, 192 sagittal slices and FOV = 256 mm × 256 mm. Diffusion-weighted images were acquired by the single-shot spin-echo echo-planar imaging (SE-EPI) sequence, using the following imaging parameters: TR/TE = 11,000/104 ms, voxel size = 2 mm × 2 mm × 2 mm, 70 contiguous axial slices, FOV = 128 × 128 mm^2^, 30 non-collinear gradient directions with a b value of 1,000 s/mm^2^ and three additional null images (*b* = 0 s/mm^2^) as reference images with NEX = 3. All the images were first visually examined to apply the following exclusion criteria: (1) data with brain lesions (unreported tumor, stroke or cyst); (2) data with artifacts and severe motion in the T_1_w images and DWIs, which would lead to ill registration and tensor fitting.

The T_1_w images were preprocessed using the FreeSurfer V5.3.0 processing stream, including the steps of registration to a template, intensity normalization, gray and white matter segmentation, tessellation of the gray/cerebrospinal fluid (CSF) and white/gray matter boundaries and cortical surface reconstruction (Fischl, 2012). DWIs were preprocessed using the FSL software V5.0.9 (Functional Magnetic Resonance Imaging of the Brain Software Library^1^). Each DWI was registered to the null image following the affine registration approach to minimize the image distortion by eddy currents and correct the subject motion (Jenkinson and Smith, 2001; Jenkinson et al., 2002; Andersson and Sotiropoulos, 2016). Notably, the subject motion could induce the alteration of the diffusion orientation, and each gradient direction of the DWIs was reoriented with the corresponding transformation matrix, which describes the rotation parameters of the subject motion (Leemans and Jones, 2009). The diffusion tensor model was fitted to calculate the voxel-wise measures of FA, MD, axial diffusivity (AD) and radial diffusivity (RD). In order to maintain the accuracy of the cross-modality image registration, the non-diffusion weighted image of each participant was skull-stripped (Smith, 2002). Using the boundary-based registration methods, each preprocessed T_1_w image was registered to the corresponding non-diffusion weighted image to perform tissue segmentation and anatomically-constrained tractography (ACT) (Greve and Fischl, 2009).

### Whole Brain Tractography

The whole-brain ACT was processed using the MRtrix3 tools (Smith et al., 2012; Tournier et al., 2019). The registered T_1_w images were segmented into five-tissue-type (5TT) format data containing the cortical gray matter, subcortical gray matter, white matter, cerebrospinal fluid and pathological tissue (Zhang et al., 2001; Smith, 2002; Smith et al., 2004; Patenaude et al., 2011). Next, fiber orientation distributions (FODs) were computed using the single-shell 3-tissue constrained spherical deconvolution (SS3T-CSD) algorithm with the DWIs and 5TT data, resulting in the response functions for the white matter, gray matter and CSF (Dhollander et al., 2019). Finally, we used the second-order integration probabilistic streamline tractography to generate whole-brain tractography with 20 seeds per voxel in each participant’s white matter mask (Tournier et al., 2010).

### Construction of Individual Corpus Callosum Connection Maps

In this work, we used identical processing pipelines to subdivide the CC into 7 and 17 segments based on its connections to the functional networks of Yeo’s atlas (Yeo et al., 2011). Firstly, individual volumetric CC masks were generated using the FreeSurfer anatomical segmentation, and the seven functionally organized networks were derived from Yeo’s atlas^2^ (Fischl et al., 2002; Yeo et al., 2011). To extract enough fibers terminated at the cortical masks, we dilated 2 mm of the generated cortical masks. Afterward, the individual CC masks and functional networks were registered to the native DWI space for tract extraction. The registration results in each step were all visually inspected and showed their accuracy. With the CC masks and functional atlases constrained, the callosal fibers that pass through the CC and connect each functional network were obtained. For each functional network, the voxels of the CC connection map were labeled by the number of callosal fibers passing through the voxel and interconnecting each functional network (Calamante et al., 2010). The MRtrix3 (Tournier et al., 2019) tools were used to perform the construction procedures for the individual CC connection maps.

### Construction of Population-Based Probabilistic Corpus Callosum Connection Maps

To construct the population-based CC connection maps, individual CC connection maps were transformed from the native DWI space to the standard Montreal Neurological Institute (MNI) space with the nearest-neighbor interpolation (Andersson et al., 2010). A CC mask of the MNI-152 T1 template was generated by the FreeSurfer software and used to construct the population-based CC connection maps by applying it to all the transformed individual CC connection maps. The connection probability was determined using Park’s approach (Park et al., 2008). For each participant, the connection probability corresponding to each network in a single voxel was defined as:


$$P\left(s,v,n\right)=\frac{\mathrm{FN}\left(s,v,n\right)}{\sum_{n=1}^{N}\mathrm{FN}\left(s,v,n\right)},$$

where *n* is the serial number of network labels (1–7), *FN*(*s*, *v*, *n*) denotes the number of streamlines connected to network *n* through voxel *v* for subject *s* from the transformed individual CC connection maps, and *P*(*s*, *v*, *n*) is the probability of the white matter connecting to networks, which is presented by the ratio of the number of streamlines connecting to network *n* to all the networks through voxel *v* for subject *s*.

**FIGURE 1:**
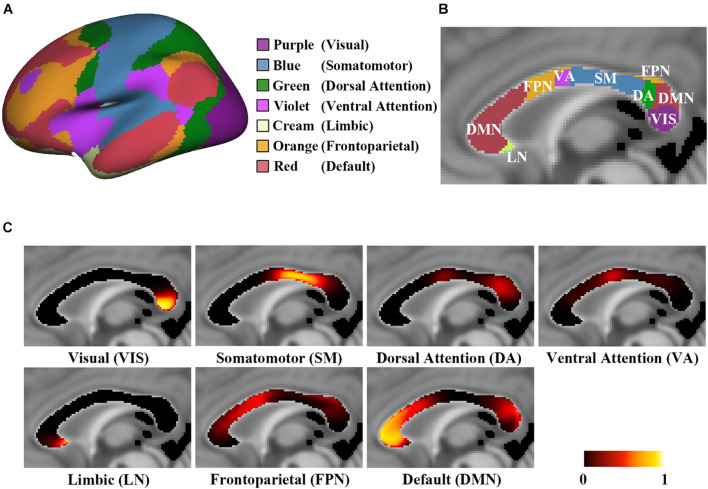
The probabilistic connection maps of 7 CC subregions. **(A)** The atlas of 7 functional networks with color-coding; **(B)** Hard segmentation map of the highest connection probability to corresponding functional networks with the same color-coding of panel **(A)**; **(C)** The probabilistic maps of the midsagittal CC, showing **P**(**v**,**n**) in each CC subregion.

For each CC voxel, the population-based connection probability to a specific network *n* was defined as:


$$P\left(v,n\right)=\frac{1}{S}\sum_{S=1}^{S}P\left(s,v,n\right),$$

where *P*(*v*, *n*) is the average connection probability of all the participants (*S* = 1,086). In the resulting group maps, the voxel value reflects the connection probability of each CC voxel interconnecting each functional network, and the population-based probabilistic CC connection maps were constructed. To reveal the highest connection probability toward multiple cortical targets in each callosal voxel, we presented the maximum likelihood labeled CC map, so-called the hard segmentation map (Figure 1 and Supplementary Figure 1; Hofer and Frahm, 2006; Chao et al., 2009).

### Reproducibility of Probabilistic Corpus Callosum Connection Maps

We examined the reproducibility of the probabilistic CC connection maps construction method by randomly separating all the participants into two age- and sex-matched groups and comparing the similarity of the resulting CC connection maps. Each group consisted of 543 participants, first group: male/female: 263/280, age = 59.11 ± 13.57 years, second group: male/female: 264/279, age = 58.99 ± 13.52 years. Following the probabilistic CC connection maps construction process, the probabilistic CC connection maps were reconstructed from the two sets. Then, two hard segmentation maps of CC were generated from both groups’ probabilistic CC connection maps. The spatial variation of the two hard segmentation maps of the CC was assessed using the Dice similarity coefficient (Dice, 1945).

### Subregional Connection Probability of Age Groups

All the participants were subdivided into seven groups by age. The groups were as follows: 73 individuals (21–30 years), 41 (31–40 years), 42 (41–50 years), 413 (51–60 years), 305 (61–70 years), 188 (71–80 years) and 24 (81–90 years), as shown in Table 1. For each subject, the mean connection probability of all the callosal voxels connecting to a specific network was defined as:


$$P\left(s,n\right)=\frac{\sum_{v=1}^{V}P\left(s,v,n\right)}{V},$$

**TABLE 1:** Demographic characteristics of seven age groups.

Variables	21–30 years	31–40 years	41–50 years	51–60 years	61–70 years	71–80 years	81–90 years	*P* value
Gender (Male/Female)	37/36	18/23	21/21	202/211	143/162	94/94	12/12	0.983
Age (Mean ± SD)	25.34 ± 2.59	34.39 ± 3.14	46.67 ± 3.09	55.77 ± 2.65	64.97 ± 2.89	74.71 ± 2.70	84.08 ± 2.08	0.000
Education (Mean ± SD)	17.40 ± 1.72	18.15 ± 3.45	13.93 ± 3.61	9.90 ± 4.33	7.87 ± 5.65	3.49 ± 4.61	4.38 ± 4.12	0.000
TIV (cm^3^) (Mean ± SD)	1,477.32 ± 127.35	1,424.89 ± 113.63	1,423.28 ± 117.98	1,410.87 ± 124.59	1,385.47 ± 121.57	1,343.54 ± 113.32	1,307.07 ± 101.67	0.000
MMSE (Mean ± SD)	29.30 ± 0.86	29.10 ± 1.09	28.19 ± 1.49	28.01 ± 1.83	26.60 ± 3.24	24.26 ± 3.76	24.63 ± 4.18	0.000

where *V* = 3,440 denotes the total voxel number in the CC masks of the MNI-152 T1 template.

### Curve Fitting of Age-Related Diffusion Indices Changes of Corpus Callosum Subregions

The probabilistic CC connection maps were transformed to the native DWI space, and the weighted mean diffusion indices *DI*(s, n) (including FA, MD, RD, and AD) of each CC subregion were calculated using the following equation (Hua et al., 2008):


$$\mathrm{DI}\left(s,n\right)=\frac{\sum \left(P_{\mathrm{trans}}\left(s,v,n\right)\times \mathrm{DI}\left(s,v\right)\right)}{\sum P_{\mathrm{trans}}\left(s,v,n\right)},$$

where *P*_*trans*_(*s*, *v*, *n*) represents the connection probability of voxel *v* in the transformed probabilistic CC connection map of network *n* for a subject *s*, and *DI*(*s*, *v*) denotes the diffusion indices (including FA, MD, RD, and AD) of voxel *v*. Each voxel *v* was extracted with an FA threshold of 0.2 to exclude the portion of gray matter or CSF (Hsu et al., 2021). The factors of the total intracranial volume (TIV), sex and years of education were regressed to control the effects on the weighted mean diffusion indices (Dufouil et al., 2003; Takao et al., 2011; Hsu et al., 2021).

For each subregion, the quadratic curve was used to model the age-related changes in each diffusion indices (including FA, MD, RD, and AD) as follows:


$$\mathrm{DI}=A\times \mathrm{age}+B\times {\mathrm{age}}^{2}+C,$$

The best-fitting model was evaluated according to the goodness of the fit with the maximally adjusted coefficient of determination (R_*adj*_^2^). Besides, the age of peak FA values and minimum MD, RD and AD values were determined when a turning point occurred in the best-fitting model.

### Statistical Analysis

Descriptive statistics of demographic data were presented as mean ± standard deviation. The Chi-square test and analysis of variance (ANOVA) were applied to compare the categorical and continuous demographic variables among the seven age groups. For each CC subregion, the connection probability differences among the seven age groups were compared using the analysis of covariance (ANCOVA) with the factors of sex, education years and TIV as covariates. Differences in the diffusion indices between the CC subregions were compared by the repeated-measures analysis of variance (RM-ANOVA) with age, sex, education years and TIV being regressed out. The Bonferroni correction for the *post hoc* test was used to control for Type I error with multiple comparisons, and a *p*-value < 0.05 was considered statistically significant. The statistical analysis was performed using the Statistical Package for Social Sciences (SPSS Version 26).

## Results

### Demographics

The demographic details of the participants from the seven age groups are shown in Table 1. Significant differences were found among the groups in terms of education years, TIV and MMSE score (*P* = 0.000, ANOVA), but not in terms of sex (Chi-square = 1.061, *P* = 0.983).

### Population-Based Probabilistic Corpus Callosum Connection Maps

The probabilistic CC connection maps toward the visual network (VIS), somatomotor network (SM), dorsal attention network (DA), ventral attention network (VA), limbic network (LN), frontoparietal network (FPN) and default mode network (DMN) are shown in Figure 1. In detail, streamlines interconnecting the VIS pass through the posterior region of the splenium streamlines interconnecting the SM pass *via* the posterior midbody and isthmus, streamlines interconnecting the DA pass *via* the ventral region of the superior splenium, streamlines interconnecting the VA pass *via* the posterior downside of the anterior midbody, streamlines interconnecting the LN pass through the rostrum, streamlines interconnecting the FPN pass through the anterior midbody, posterior region of the rostral body and the upper region of the superior splenium, and streamlines interconnecting the DMN pass through the genu, anterior region of the rostral body and dorsal region of the superior splenium. The Dice coefficient of two hard segmentation maps of 7 CC subregions was 0.9849, and that of 17 CC subregions was 0.9817.

### Differences in Diffusion Indices Between Corpus Callosum Subregions

**FIGURE 2:**
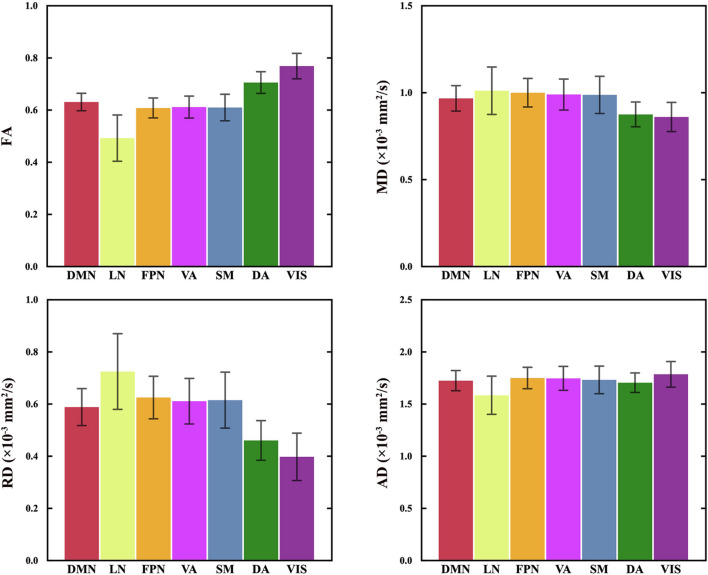
The diffusion indices of 7 CC subregions. The mean and standard deviation of the regressed **D***I*(**s**,**n**) of all participants are plotted in the order of the spatial locations of all CC subregions, generally from the anterior to posterior part in the CC. RM-ANOVA showed significant differences in FA, MD, AD and RD between the CC subregions. FA values of the CC subregion connecting to VIS and DA are statistically higher than other subregions. MD and RD values of the CC subregions connecting to VIS and DA are statistically lower than those of other subregions. See Supplementary Table 1 for the details of the statistical analysis of paired comparisons. VIS, CC subregion connecting to the visual network; SM, CC subregion connecting to the somatomotor network; DA, CC subregion connecting to the dorsal attention network; VA, CC subregion connecting to the ventral attention network; LN, CC subregion connecting to the limbic network; FPN, CC subregion connecting to the frontoparietal network; DMN, CC subregion connecting to the default mode network; FA, fractional anisotropy; MD, mean diffusivity; RD, radial diffusivity; AD, axial diffusivity.

Figure 2 shows the mean diffusion indices of seven CC subregions. Significant differences were found in FA, MD, AD and RD between the CC subregions (*P* = 0.000, RM-ANOVA). The FA values of the subregions to VIS and DA (range 0.7–0.8) were significantly higher than other subregions (range 0.5–0.6, *P* < 0.05, Bonferroni corrected). On the other hand, the mean MD and RD values of the subregions to VIS and DA (MD: 0.8–0.9 × 10^–3^ mm^2^/s; RD: 0.4–0.5 × 10^–3^ mm^2^/s) were lower than other subregions (MD: 0.9–1 × 10^–3^ mm^2^/s; RD: 0.6–0.7 × 10^–3^ mm^2^/s, *P* < 0.05, Bonferroni corrected). No significant difference was found in MD between the subregions to SM and VA (*P* = 1.000, Bonferroni corrected), LN and FPN (*P* = 0.065, Bonferroni corrected); no significant difference in FA between the subregions to SM and VA (*P* = 1.000, Bonferroni corrected), SM and FPN (*P* = 1.000, Bonferroni corrected); no significant difference in AD between the subregions to SM and DMN (*P* = 0.895, Bonferroni corrected), VA and FPN (*P* = 0.617, Bonferroni corrected); no significant difference in RD between the subregions to SM and VA (*P* = 0.480, Bonferroni corrected). The details of pairwise comparisons in diffusion indices between the CC subregions by Bonferroni *post hoc* test can be found in Supplementary Table 1.

### Subregional Connection Probability Differences Between Age Groups

**FIGURE 3:**
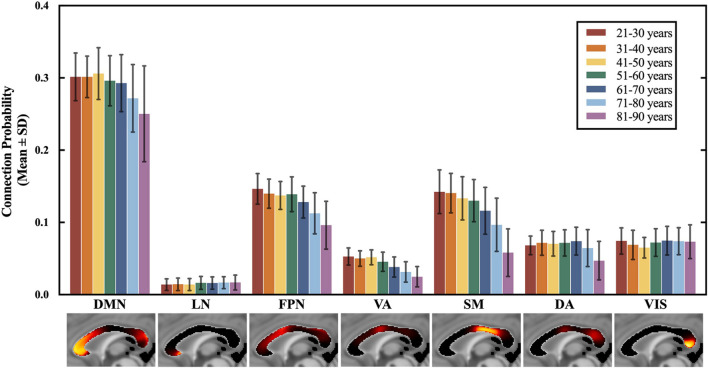
The connection probability of 7 CC subregions across all 7 age groups. The mean and standard deviation of the regressed **P**(**s**,**n**) of each age group are plotted in the order of the spatial locations of all CC subregions, generally from the anterior to posterior part in the CC. ANCOVA showed significant differences among the 7 age groups for the subregions to DMN, FPN, VA, SM, DA, and VIS, which exhibited an anterior-to-posterior changing gradient. The subregions to DMN, FPN, VA and SM that are located in the anterior and middle part of the CC showed an earlier and severer decrease in the connection probability than the subregion to DA and VIS in the posterior CC. Statistical analysis of the paired comparisons is shown in Supplementary Table 2. VIS, CC subregion connecting to the visual network; SM, CC subregion connecting to the somatomotor network; DA, CC subregion connecting to the dorsal attention network; VA, CC subregion connecting to the ventral attention network; LN, CC subregion connecting to the limbic network; FPN, CC subregion connecting to the frontoparietal network; DMN, CC subregion connecting to the default mode network.

The connection probability of the seven CC subregions across age groups is shown in Figure 3. Significant subregional connection probability differences were found among the seven age groups in the subregions interconnecting VIS, SM, DA, VA, FPN and DMN (*P* = 0.000, ANCOVA), but no significant difference was found in LN (*P* = 0.138, ANCOVA). Specifically, the subregional connection probability of the subregions to SM, VA, FPN and DMN showed significant decreases in the groups older than 60 years (*P* < 0.05, Bonferroni corrected), and the connection probability of the subregion to DA only showed significant decreases in the groups older than 70 years (*P* < 0.05, Bonferroni corrected). On the contrary, participants aged 61–70 years had a larger connection probability than those in the 41–50 years group in the subregion to VIS (*P* < 0.05, Bonferroni corrected). Similar comparison results were found between the CC subregions to 17 subnetworks. The details of pairwise comparisons of connection probability between age groups by Bonferroni *post hoc* test can be found in Supplementary Tables 2, 3.

### Curve Fitting of Age-Related Diffusion Indices Changes of Corpus Callosum Subregions

**FIGURE 4:**
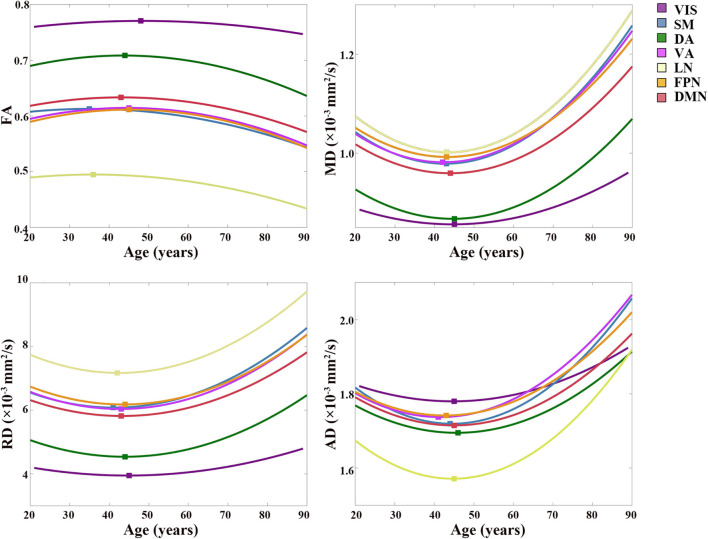
Best-fitting curves for regressed diffusion indices vs. age. Ages of peak FA values and minimum MD, RD and AD values from the quadratic fits are labeled by colored squares for all the subregions. Fit curves of all diffusion indices in the subregion to VIS are flat, which indicated that slight changes occurred in the microstructure of the CC fibers interconnecting the VIS during the aging process. On the contrary, fit curves of the rest subregions exhibited a U shape of the fit curves of MD, RD, and AD, and an inverted U shape of the fit curve of FA. The scatter plots of DTI indices vs. age are shown in Supplementary Figure 3. VIS, CC subregion connecting to the visual network; SM, CC subregion connecting to the somatomotor network; DA, CC subregion connecting to the dorsal attention network; VA, CC subregion connecting to the ventral attention network; LN, CC subregion connecting to the limbic network; FPN, CC subregion connecting to the frontoparietal network; DMN, CC subregion connecting to the default mode network; FA, fractional anisotropy; MD, mean diffusivity; RD, radial diffusivity; AD, axial diffusivity.

The age-related FA changing trajectories of all the CC subregions correspond to small *R*^2^ values and *R*_*adj*_^2^ values within the range of 0.006–0.091 (Table 2). These fit curves show that the FA slightly increased during early adulthood and then decreased through later adulthood (Figure 4 and Supplementary Figure 3). The ages of peak FA values varied from 35 years of the subregion DA to 50 years of the subregion VIS (Figure 4).

**TABLE 2:** Fitting parameters for quadratic fit equations of each DTI parameter.

FA	Intercept	Linear term (×10^–2^)	Quadratic term (×10^–4^)	*R* ^2^	*R*^2^ adjust	*P* value
VIS	0.737	0.138	−0.143	0.008	0.006	0.000
SM	0.585	0.160	−0.229	0.059	0.057	0.000
DA	0.644	0.294	−0.337	0.083	0.082	0.000
VA	0.550	0.291	−0.326	0.070	0.068	0.000
LN	0.468	0.151	−0.210	0.016	0.014	0.000
FPN	0.541	0.312	−0.344	0.088	0.086	0.000
DMN	0.581	0.244	−0.283	0.093	0.091	0.000

**MD**	**Intercept (×10^–3^)**	**Linear term (×10^–5^)**	**Quadratic term (×10^–7^)**	***R* ^2^**	***R*^2^ adjust**	***P* value**

VIS	0.962	−0.474	0.531	0.047	0.046	0.000
SM	1.205	−1.060	1.243	0.170	0.168	0.000
DA	1.062	−0.873	0.979	0.196	0.194	0.000
VA	1.188	−0.980	1.162	0.212	0.210	0.000
LN	1.250	−1.145	1.318	0.117	0.115	0.000
FPN	1.196	−0.941	1.089	0.203	0.201	0.000
DMN	1.156	−0.894	1.017	0.209	0.207	0.000

**RD**	**Intercept (×10^–3^)**	**Linear term (×10^–5^)**	**Quadratic term (×10^–7^)**	***R* ^2^**	***R*^2^ adjust**	***P* value**

VIS	0.480	−0.383	0.429	0.027	0.025	0.000
SM	0.787	−0.866	1.051	0.141	0.140	0.000
DA	0.629	−0.800	0.911	0.163	0.161	0.000
VA	0.794	−0.891	1.044	0.177	0.175	0.000
LN	0.921	−0.962	1.133	0.085	0.084	0.000
FPN	0.810	−0.883	1.014	0.179	0.178	0.000
DMN	0.754	−0.794	0.917	0.194	0.193	0.000

**AD**	**Intercept (×10^–3^)**	**Linear term (×10^–5^)**	**Quadratic term (×10^–7^)**	***R* ^2^**	***R*^2^ adjust**	***P* value**

VIS	1.926	−0.655	0.734	0.042	0.040	0.000
SM	2.041	−1.447	1.627	0.164	0.162	0.000
DA	1.927	−1.018	1.115	0.142	0.141	0.000
VA	1.977	−1.157	1.397	0.198	0.196	0.000
LN	1.908	−1.510	1.690	0.097	0.095	0.000
FPN	1.967	−1.058	1.241	0.181	0.179	0.000
DMN	1.961	−1.093	1.217	0.165	0.164	0.000

*Parameters for the quadratic equation are shown, including intercept, linear and quadratic terms for all seven regions of the corpus callosum, as well as the R^2^ and R^2^ adjust value and p value for the entire fit.*

*The unit of MD, AD, and RD is mm^2^/s.*

*VIS, CC subregion connecting to the visual network; SM, CC subregion connecting to the somatomotor network; DA, CC subregion connecting to the dorsal attention network; VA, CC subregion connecting to the ventral attention network; LN, CC subregion connecting to the limbic network; FPN, CC subregion connecting to the frontoparietal network; DMN, CC subregion connecting to the default mode network; FA, fractional anisotropy; MD, mean diffusivity; RD, radial diffusivity; AD, axial diffusivity.*

Except for the fit curves of MD, RD and AD of the subregion to VIS (R_*adj*_^2^: 0.025–0.046) and LN (R_*adj*_^2^: 0.084–0.115) that had lower R_*adj*_^2^, the fit curves of other subregions showed fit goodness with higher R_*adj*_^2^ values in all the diffusion indices (R_*adj*_^2^ > 0.1, Table 2). The changing trends of MD, RD and AD were opposite to that of FA, as they slightly decreased through young adulthood and then increased over 0.0002 mm^2^/s than its initial decrease, and their increases of the subregion to VIS were smaller than other subregions (Figure 4 and Supplementary Figure 3). The ages of minimum MD, RD and AD of each CC subregion showed a slight variance (Figure 4).

## Discussion

This study presented a population-based probabilistic topography of the CC related to functionally organized networks, providing a comprehensive description of the interconnections within the visual, sensory-motor and distributed networks in the higher-order association cortex and revealing the functional specialization of the CC subregions. Based on the functional CC subregions, we found region-specific aging patterns exhibited in the CC. Besides, the subregions interconnecting the SM, VA, FPN and DMN showed severer alterations compared with other CC subregions in terms of the structural connectivity and microstructure. This study revealed the callosal subregions related to functional networks and uncovered the overall “anterior-to-posterior” changing trend during the aging process.

The population-based probabilistic CC connection maps were verified high reproducibility. The spatial localization of each CC subregion was close to the location of the functional networks it connected and following the former geometric-based and tractography-based CC parcellations (Witelson, 1989; Chao et al., 2009; Lebel et al., 2010; van der Knaap and van der Ham, 2011). The spatial locations of the CC subregions were consistent with the stimulus-induced activations in the CC, such as the motor, tactile, visual, auditory, gustatory and memory tasks (Mazerolle et al., 2008; Yarkoni et al., 2009; Fabri et al., 2011). For instance, these activations in the CC were distributed according to the anterior (taste stimuli), middle (motor task), middle and posterior (tactile stimuli) and splenium (visual stimuli) areas (Fabri et al., 2011). According to the CC connection maps, the subregion interconnecting VIS also projected fibers to DA and DMN (Figure 1C). The visual system processes information either through the dorsal stream or the ventral stream. The parietal areas (part of the DA) are critical parts of the dorsal stream to process the visual information of spatial relationships and control spatially directed actions. The temporal cortex (part of the DMN) is a critical part of the ventral stream to process subject recognition information (Mishkin et al., 1983; Milner and Goodale, 2006). Our findings revealed the underlying mechanism of the dorsal and ventral stream in the cerebral network system. Moreover, based on the probabilistic CC connection maps of 17 subnetworks, the CC subregions interconnecting the subnetworks that belong to VIS showed different connection probabilities (Supplementary Figure 1; Sacchet et al., 2016). This result indicated the dominant interhemispheric communication transferring area in VIS *via* the CC, which may provide the structural foundation of the difference in the functional connectivity within the local networks (Yeo et al., 2011).

Significant differences in the DTI indices were found between the CC subregions. Posterior subregions interconnecting VIS and DA showed higher mean FA, AD and lower mean MD, RD compared with the other CC subregions, whereas the anterior subregion interconnecting the LN showed minimum mean FA, AD, and maximum mean MD, RD (Figure 2). Different parameters reflect different aspects of white matter microstructure. Specifically, FA and MD measure the diffusion barrier to water molecules, AD and RD measures the parallel and perpendicular diffusivities (Basser and Pierpaoli, 1996; Rogalski et al., 2012). According to the fiber composition in CC, the anterior part of CC concentrated low myelinated, small-caliber, slow-conducting CC fibers, while the posterior part contained highly-myelinated, large-caliber, fast-conducting fibers (Aboitiz et al., 1992a, 2003). Previous studies found a positive correlation between FA and the conduction velocity in the CC, which is related to the myelination or axon diameter (Caminiti et al., 2013). The greater the anisotropy, the more directional and linear the diffusion of water molecules. Therefore, the microstructural differences between the subregions are consistent with the fiber composition in the CC.

Significant decreases in the connection probability were found between the younger and older age groups in the CC subregions to DMN, FPN, VA, and SM (Figure 3), which indicated an age-related decrease in the structural connectivity between the corresponding functional networks. However, the connection probability of the subregion to DA started to decrease at a very late age, and the connection probability of the subregion to VIS slightly increased with age (Figure 3). The changes in the CC subregion to VIS were consistent with previous findings reporting that the volume of the CC subregions connecting to the occipital lobe was increased with age (Lebel et al., 2010). According to the CC connection maps, the CC subregions located in the anterior and middle CC showed a larger decrease in the connection probability than the subregions in the posterior CC during the aging process. This finding was following the anterior-to-posterior changing trend of the CC reported by previous research (Salat et al., 2005; Bucur et al., 2008; Davis et al., 2009; Lebel et al., 2010).

The diffusion indices of the CC subregions also exhibited an anterior-to-posterior changing trend, and severer changes were found in the CC subregions to DMN, FPN, VA, and SM compared with the CC subregions to DA and VIS (Figure 4). This result was consistent with previous findings that the differences in DTI indices between younger and older adults were more pronounced in the anterior corpus callosum than in the posterior region (Salat et al., 2005; Bucur et al., 2008; Davis et al., 2009; Lebel et al., 2010). Specific age-related changes in DTI indices reflect different aspects of white matter microstructure. The greater water content associated with atrophy in the aging brain could cause the increase of AD and MD, and the loss of myelinated fibers and decreased axonal density would result in the increase of RD and lower FA (Scheltens et al., 1995; Moseley, 2002).

The observed region-specific changing patterns in the connection probability and diffusion indices indicated that the CC subregions exhibited different aging patterns. We may speculate two aspects that account for this finding. On the one hand, the “last in, first out” hypothesis of aging posits that the cortical regions are not equally affected during aging, and late-maturing regions are preferentially vulnerable to age (Raz, 1999; Grieve et al., 2005; Fjell et al., 2009). Therefore, the region-specific aging patterns in the CC subregions are associated with the different atrophy of the connected cortical targets. We found that the CC subregion to SM exhibited severer and earlier changes in the diffusion indices with age. Similarly, it was shown by age-related structural studies that reduction in volume/size and cortical thinning occurred in the primary sensory-motor cortices in the elderly (Haug and Eggers, 1991; Salat et al., 2004; McGinnis et al., 2011). On the other hand, previous studies reported that the anterior CC fibers myelinate much later in normal development, while the posterior CC fibers myelinate early (Aboitiz et al., 1992a, 2003). Therefore, the CC fibers in each subregion followed the “last in, first out” hypothesis and exhibited distinct aging patterns.

This study has several limitations. Firstly, due to the intrinsic limitation of DTI in the regions with complex fiber heterogeneity, we used the SS3T-CSD algorithm to estimate the fiber orientation distributions for whole-brain tractography. We applied the most used DTI measurements to study the aging pattern in the CC subregions. Advanced diffusion models with multi-shell protocol should be used to capture the microstructure (Raffelt et al., 2017). Secondly, the CC masks were generated by segmenting the T1 images and registered to the DWI images, the multi-modal registration is challenging because DTI is susceptible to both affine/linear (e.g., eddy-current and head motion) and non-linear echo planar image field distortions. The registration results in each step were all visually inspected and showed their accuracy. Finally, there were relatively small samples in groups from 21–50 years and 81–90 years, which may bias the results for our imaging measures. In this work, we tried to analyze as much as possible data from our center and therefore we combined two imaging datasets with identical imaging scanner and protocols. Further study should recruit more participants to explore the microstructural changes of the CC during the earlier lifespan.

## Conclusion

The current study provided a reliable tractography-based CC functional topography, which presented the spatial location of the callosal subregions interconnecting the functional networks. This functional topography of the CC is essential to understand the white matter organization in primary and higher-order functional systems and is also helpful in planning corpus callosotomy. We showed that the CC underwent an anterior-to-posterior changing trend during the aging process, with the CC subregions interconnecting the SM, VA, FPN, and DMN showing severer alterations compared with other CC subregions. This finding revealed the aging pattern of the CC subregions and provided a baseline to identify the presence and timing of callosal abnormalities in various brain disorders.

## Data Availability Statement

The data analyzed in this study is subject to the following licenses/restrictions: The datasets presented in this article are not readily available because the datasets containing information that could compromise research participant privacy/consent. Requests to access these datasets should be directed to C-PL, chingpolin@gmail.com.

## Ethics Statement

The studies involving human participants were reviewed and approved by the Institutional Review Board of Taipei Veterans General Hospital and the Institutional Review Board of National Yang-Ming University. The patients/participants provided their written informed consent to participate in this study.

## Author Contributions

YL: study design and concept, image processing, data analysis and interpretation, bibliography research, and drafting of the manuscript. C-CHH: MRI acquisition, image processing, revising the manuscript, and approving the submitted version. C-CH: study design and concept, MRI acquisition, image processing, data interpretation, and approving the submitted version. YZ, JZ, S-JT, and L-KC: study concept, revising the manuscript, and approving the submitted version. C-PL and C-YL: study design and concept, data interpretation, bibliography research, critical revision of the manuscript, and approving the submitted version. All authors contributed to the article and approved the submitted version.

## Conflict of Interest

The authors declare that the research was conducted in the absence of any commercial or financial relationships that could be construed as a potential conflict of interest.

## Publisher’s Note

All claims expressed in this article are solely those of the authors and do not necessarily represent those of their affiliated organizations, or those of the publisher, the editors and the reviewers. Any product that may be evaluated in this article, or claim that may be made by its manufacturer, is not guaranteed or endorsed by the publisher.

## Footnotes

1
https://fsl.fmrib.ox.ac.uk/fsl/fslwiki/FSL

2
http://surfer.nmr.mgh.harvard.edu/fswiki/CorticalParcellation_Yeo2011
