# Supplementary material for: Connectivity-Based Topographical Changes of the Corpus Callosum During Aging
Source: Front Aging Neurosci. 2021 Oct 20;13:753236. doi: 10.3389/fnagi.2021.753236 (PMC8565522; doi:10.3389/fnagi.2021.753236)
Supplement: Supplementary file 1 [file Data_Sheet_1.docx]

Supplementary Material

**Supplementary Figure 1.** The probabilistic connection maps of 17 CC subregions

**Supplementary Figure 2.** The connection probability of 17 CC subregions across all 7 age groups

**Supplementary Figure 3.** Diffusion indices vs. age

**Supplementary Table 1.** The comparison of diffusion indices between 7 CC subregions with Bonferroni-corrected post hoc tests

**Supplementary Table 2.** The comparison of connection probability between age groups for 7 CC subregions with Bonferroni-corrected post hoc tests

**Supplementary Table 3.** The comparison of connection probability between age groups for 17 CC subregions with Bonferroni-corrected post hoc tests

**
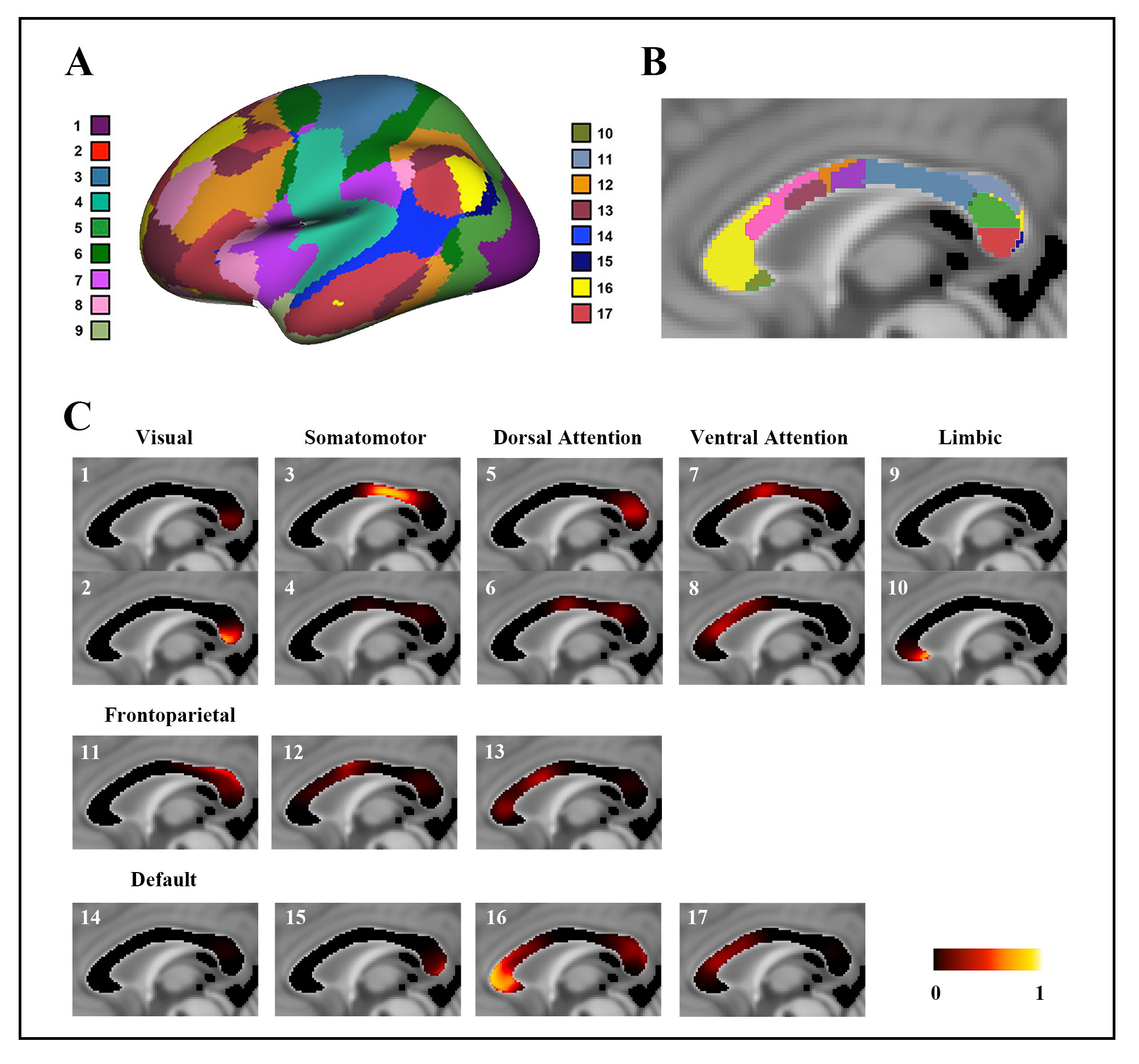
**

**Supplementary Figure 1. The probabilistic connection maps of 17 CC subregions.** (A) The atlas of 17 functional networks with color-coding; (B) Hard segmentation map of the highest connection probability to corresponding functional networks with the same color-coding of (A); (C) The probabilistic map of the midsagittal CC, showing $\boldsymbol{P}(\boldsymbol{v},\boldsymbol{n})$ in each CC subregion. The labels with the corresponding 17 subnetworks were derived by Sacchet et al. (Sacchet et al., 2016).

**
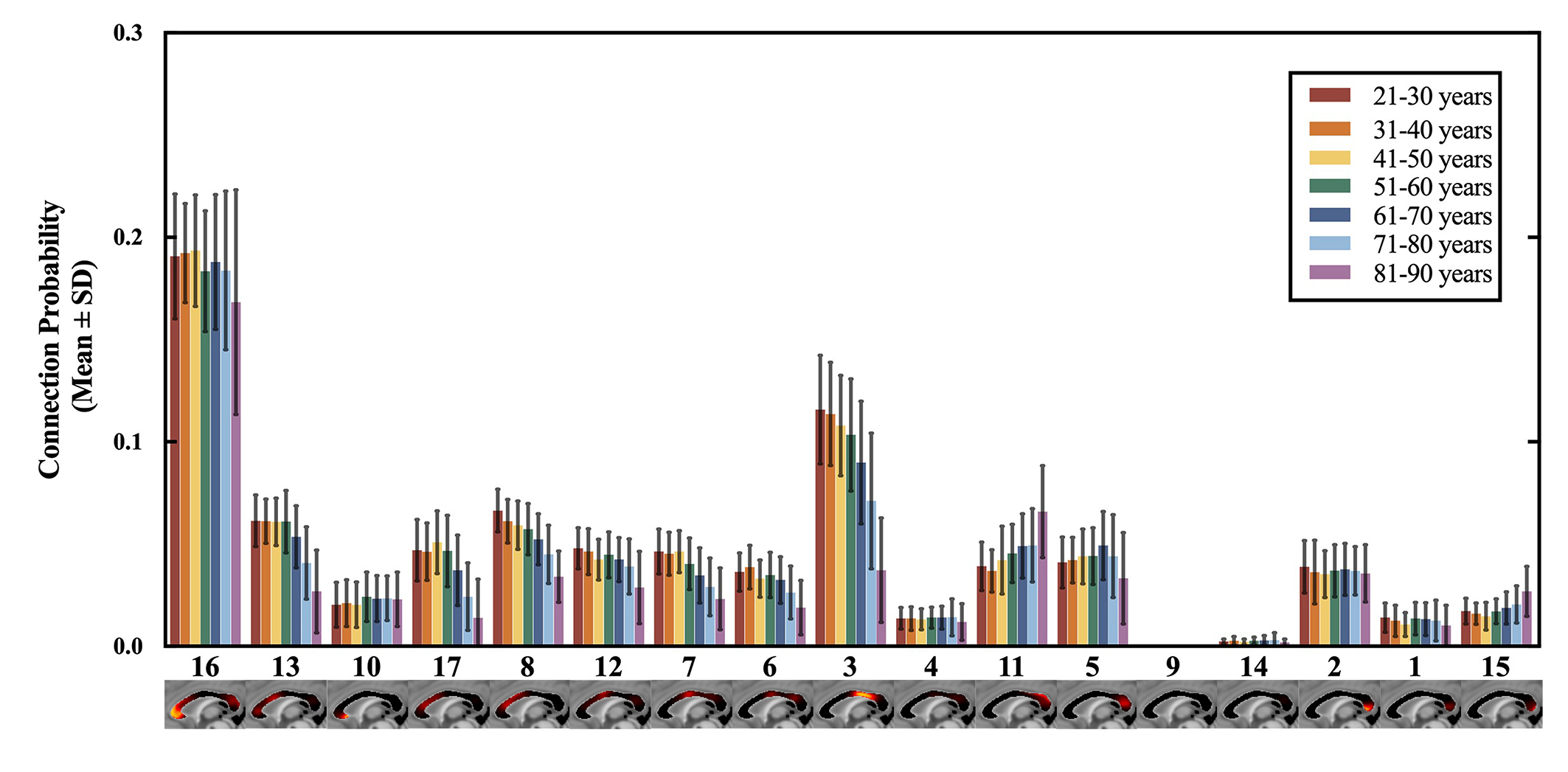
**

**Supplementary Figure 2. The connection probability of 17 CC subregions across all 7 age groups.** The mean and standard deviation of the regressed $\boldsymbol{P}\left( \boldsymbol{s},\boldsymbol{n} \right)$ of each age group are plotted in the order of the spatial locations of all CC subregions, generally from the anterior to posterior part in the CC. ANCOVA showed significant differences between the 7 age groups for most subregions except for subregions 1, 2, 4, 10, 14, and 16, which exhibited an anterior-to-posterior changing gradient. Statistical analysis of the paired comparisons is shown in Supplementary Table 3.


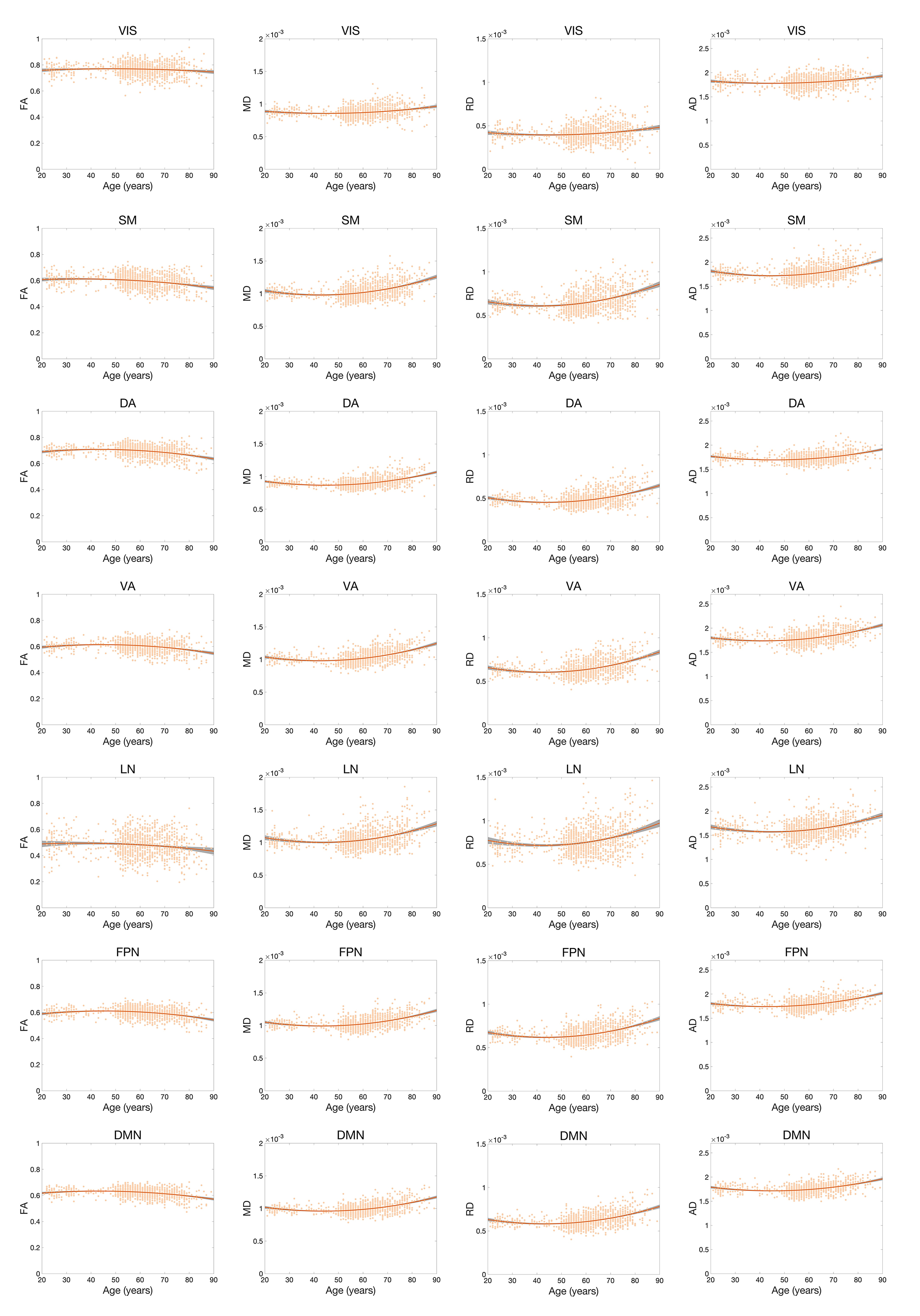


**Supplementary Figure 3. Diffusion indices vs. age.** Regressed FA, MD, RD and AD vs. age plots (orange dots) and the best-fit curves (orange lines) with a 95% confidence interval are shown for each CC subregion. The unit of MD, AD, and RD is mm/s. VIS, CC subregion connecting to the visual network; SM, CC subregion connecting to the somatomotor network; DA, CC subregion connecting to the dorsal attention network; VA, CC subregion connecting to the ventral attention network; LN, CC subregion connecting to the limbic network; FPN, CC subregion connecting to the frontoparietal network; DMN, CC subregion connecting to the default mode network.

**Supplementary Table 1. The comparison of diffusion indices between 7 CC subregions with Bonferroni-corrected post hoc tests**

| FA | Comparison | Mean difference | Standard error | P-value |
| --- | --- | --- | --- | --- |
| VIS | SM | 0.159 | 0.002 | *0.000* |
|  | DA | 0.063 | 0.001 | *0.000* |
|  | VA | 0.157 | 0.002 | *0.000* |
|  | LN | 0.276 | 0.003 | *0.000* |
|  | FPN | 0.160 | 0.002 | *0.000* |
|  | DMN | 0.138 | 0.001 | *0.000* |
| SM | VIS | -0.159 | 0.002 | *0.000* |
|  | DA | -0.096 | 0.001 | *0.000* |
|  | VA | -0.001 | 0.001 | 1.000 |
|  | LN | 0.117 | 0.003 | *0.000* |
|  | FPN | 0.002 | 0.001 | 1.000 |
|  | DMN | -0.021 | 0.001 | *0.000* |
| DA | VIS | -0.063 | 0.001 | *0.000* |
|  | SM | 0.096 | 0.001 | *0.000* |
|  | VA | 0.094 | 0.001 | *0.000* |
|  | LN | 0.213 | 0.003 | *0.000* |
|  | FPN | 0.098 | 0.001 | *0.000* |
|  | DMN | 0.075 | 0.001 | *0.000* |
| VA | VIS | -0.157 | 0.002 | *0.000* |
|  | SM | 0.001 | 0.001 | 1.000 |
|  | DA | -0.094 | 0.001 | *0.000* |
|  | LN | 0.119 | 0.003 | *0.000* |
|  | FPN | 0.003 | 0.001 | *0.000* |
|  | DMN | -0.020 | 0.001 | *0.000* |
| LN | VIS | -0.276 | 0.003 | *0.000* |
|  | SM | -0.117 | 0.003 | *0.000* |
|  | DA | -0.213 | 0.003 | *0.000* |
|  | VA | -0.119 | 0.003 | *0.000* |
|  | FPN | -0.116 | 0.003 | *0.000* |
|  | DMN | -0.138 | 0.002 | *0.000* |
| FPN | VIS | -0.160 | 0.002 | *0.000* |
|  | SM | -0.002 | 0.001 | 1.000 |
|  | DA | -0.098 | 0.001 | *0.000* |
|  | VA | -0.003 | 0.001 | *0.000* |
|  | LN | 0.116 | 0.003 | *0.000* |
|  | DMN | -0.023 | 0.001 | *0.000* |
| DMN | VIS | -0.138 | 0.001 | *0.000* |
|  | SM | 0.021 | 0.001 | *0.000* |
|  | DA | -0.075 | 0.001 | *0.000* |
|  | VA | 0.020 | 0.001 | *0.000* |
|  | LN | 0.138 | 0.002 | *0.000* |
|  | FPN | 0.023 | 0.001 | *0.000* |
| MD | Comparison | Mean difference | Standard error | P-value |
| VIS | SM | 0.000 | 0.000 | *0.000* |
|  | DA | 0.000 | 0.000 | *0.000* |
|  | VA | 0.000 | 0.000 | *0.000* |
|  | LN | 0.000 | 0.000 | *0.000* |
|  | FPN | 0.000 | 0.000 | *0.000* |
|  | DMN | 0.000 | 0.000 | *0.000* |
| SM | VIS | 0.000 | 0.000 | *0.000* |
|  | DA | 0.000 | 0.000 | *0.000* |
|  | VA | 0.000 | 0.000 | 1.000 |
|  | LN | 0.000 | 0.000 | *0.000* |
|  | FPN | 0.000 | 0.000 | *0.000* |
|  | DMN | 0.000 | 0.000 | *0.000* |
| DA | VIS | 0.000 | 0.000 | *0.000* |
|  | SM | 0.000 | 0.000 | *0.000* |
|  | VA | 0.000 | 0.000 | *0.000* |
|  | LN | 0.000 | 0.000 | *0.000* |
|  | FPN | 0.000 | 0.000 | *0.000* |
|  | DMN | 0.000 | 0.000 | *0.000* |
| VA | VIS | 0.000 | 0.000 | *0.000* |
|  | SM | 0.000 | 0.000 | 1.000 |
|  | DA | 0.000 | 0.000 | *0.000* |
|  | LN | 0.000 | 0.000 | *0.000* |
|  | FPN | 0.000 | 0.000 | *0.000* |
|  | DMN | 0.000 | 0.000 | *0.000* |
| LN | VIS | 0.000 | 0.000 | *0.000* |
|  | SM | 0.000 | 0.000 | *0.000* |
|  | DA | 0.000 | 0.000 | *0.000* |
|  | VA | 0.000 | 0.000 | *0.000* |
|  | FPN | 0.000 | 0.000 | 0.065 |
|  | DMN | 0.000 | 0.000 | *0.000* |
| FPN | VIS | 0.000 | 0.000 | *0.000* |
|  | SM | 0.000 | 0.000 | *0.000* |
|  | DA | 0.000 | 0.000 | *0.000* |
|  | VA | 0.000 | 0.000 | *0.000* |
|  | LN | 0.000 | 0.000 | 0.065 |
|  | DMN | 0.000 | 0.000 | *0.000* |
| DMN | VIS | 0.000 | 0.000 | *0.000* |
|  | SM | 0.000 | 0.000 | *0.000* |
|  | DA | 0.000 | 0.000 | *0.000* |
|  | VA | 0.000 | 0.000 | *0.000* |
|  | LN | 0.000 | 0.000 | *0.000* |
|  | FPN | 0.000 | 0.000 | *0.000* |
| RD | Comparison | Mean difference | Standard error | P-value |
| VIS | SM | 0.000 | 0.000 | *0.000* |
|  | DA | 0.000 | 0.000 | *0.000* |
|  | VA | 0.000 | 0.000 | *0.000* |
|  | LN | 0.000 | 0.000 | *0.000* |
|  | FPN | 0.000 | 0.000 | *0.000* |
|  | DMN | 0.000 | 0.000 | *0.000* |
| SM | VIS | 0.000 | 0.000 | *0.000* |
|  | DA | 0.000 | 0.000 | *0.000* |
|  | VA | 0.000 | 0.000 | 0.480 |
|  | LN | 0.000 | 0.000 | *0.000* |
|  | FPN | 0.000 | 0.000 | *0.000* |
|  | DMN | 0.000 | 0.000 | *0.000* |
| DA | VIS | 0.000 | 0.000 | *0.000* |
|  | SM | 0.000 | 0.000 | *0.000* |
|  | VA | 0.000 | 0.000 | *0.000* |
|  | LN | 0.000 | 0.000 | *0.000* |
|  | FPN | 0.000 | 0.000 | *0.000* |
|  | DMN | 0.000 | 0.000 | *0.000* |
| VA | VIS | 0.000 | 0.000 | *0.000* |
|  | SM | 0.000 | 0.000 | 0.480 |
|  | DA | 0.000 | 0.000 | *0.000* |
|  | LN | 0.000 | 0.000 | *0.000* |
|  | FPN | 0.000 | 0.000 | *0.000* |
|  | DMN | 0.000 | 0.000 | *0.000* |
| LN | VIS | 0.000 | 0.000 | *0.000* |
|  | SM | 0.000 | 0.000 | *0.000* |
|  | DA | 0.000 | 0.000 | *0.000* |
|  | VA | 0.000 | 0.000 | *0.000* |
|  | FPN | 0.000 | 0.000 | *0.000* |
|  | DMN | 0.000 | 0.000 | *0.000* |
| FPN | VIS | 0.000 | 0.000 | *0.000* |
|  | SM | 0.000 | 0.000 | *0.000* |
|  | DA | 0.000 | 0.000 | *0.000* |
|  | VA | 0.000 | 0.000 | *0.000* |
|  | LN | 0.000 | 0.000 | *0.000* |
|  | DMN | 0.000 | 0.000 | *0.000* |
| DMN | VIS | 0.000 | 0.000 | *0.000* |
|  | SM | 0.000 | 0.000 | *0.000* |
|  | DA | 0.000 | 0.000 | *0.000* |
|  | VA | 0.000 | 0.000 | *0.000* |
|  | LN | 0.000 | 0.000 | *0.000* |
|  | FPN | 0.000 | 0.000 | *0.000* |
| AD | Comparison | Mean difference | Standard error | P-value |
| VIS | SM | 0.000 | 0.000 | *0.000* |
|  | DA | 0.000 | 0.000 | *0.000* |
|  | VA | 0.000 | 0.000 | *0.000* |
|  | LN | 0.000 | 0.000 | *0.000* |
|  | FPN | 0.000 | 0.000 | *0.000* |
|  | DMN | 0.000 | 0.000 | *0.000* |
| SM | VIS | 0.000 | 0.000 | *0.000* |
|  | DA | 0.000 | 0.000 | *0.000* |
|  | VA | 0.000 | 0.000 | *0.000* |
|  | LN | 0.000 | 0.000 | *0.000* |
|  | FPN | 0.000 | 0.000 | *0.000* |
|  | DMN | 0.000 | 0.000 | 0.895 |
| DA | VIS | 0.000 | 0.000 | *0.000* |
|  | SM | 0.000 | 0.000 | *0.000* |
|  | VA | 0.000 | 0.000 | *0.000* |
|  | LN | 0.000 | 0.000 | *0.000* |
|  | FPN | 0.000 | 0.000 | *0.000* |
|  | DMN | 0.000 | 0.000 | *0.000* |
| VA | VIS | 0.000 | 0.000 | *0.000* |
|  | SM | 0.000 | 0.000 | *0.000* |
|  | DA | 0.000 | 0.000 | *0.000* |
|  | LN | 0.000 | 0.000 | *0.000* |
|  | FPN | 0.000 | 0.000 | 0.617 |
|  | DMN | 0.000 | 0.000 | *0.000* |
| LN | VIS | 0.000 | 0.000 | *0.000* |
|  | SM | 0.000 | 0.000 | *0.000* |
|  | DA | 0.000 | 0.000 | *0.000* |
|  | VA | 0.000 | 0.000 | *0.000* |
|  | FPN | 0.000 | 0.000 | *0.000* |
|  | DMN | 0.000 | 0.000 | *0.000* |
| FPN | VIS | 0.000 | 0.000 | *0.000* |
|  | SM | 0.000 | 0.000 | *0.000* |
|  | DA | 0.000 | 0.000 | *0.000* |
|  | VA | 0.000 | 0.000 | 0.617 |
|  | LN | 0.000 | 0.000 | *0.000* |
|  | DMN | 0.000 | 0.000 | *0.000* |
| DMN | VIS | 0.000 | 0.000 | *0.000* |
|  | SM | 0.000 | 0.000 | 0.895 |
|  | DA | 0.000 | 0.000 | *0.000* |
|  | VA | 0.000 | 0.000 | *0.000* |
|  | LN | 0.000 | 0.000 | *0.000* |
|  | FPN | 0.000 | 0.000 | *0.000* |

VIS, CC subregion connecting to the visual network; SM, CC subregion connecting to the somatomotor network; DA, CC subregion connecting to the dorsal attention network; VA, CC subregion connecting to the ventral attention network; LN, CC subregion connecting to the limbic network; FPN, CC subregion connecting to the frontoparietal network; DMN, CC subregion connecting to the default mode network.

**Supplementary Table 2. The comparison of connection probability between age groups for 7 CC subregions with Bonferroni-corrected post hoc tests**

| VIS | Comparison | Mean difference | Standard error | P-value |
| --- | --- | --- | --- | --- |
| 21-30 years | 31-40 years | 0.005 | 0.004 | 1.000 |
|  | 41-50 years | 0.009 | 0.004 | 0.236 |
|  | 51-60 years | 0.002 | 0.002 | 1.000 |
|  | 61-70 years | 0.000 | 0.002 | 1.000 |
|  | 71-80 years | 0.000 | 0.003 | 1.000 |
|  | 81-90 years | 0.001 | 0.005 | 1.000 |
| 31-40 years | 21-30 years | -0.005 | 0.004 | 1.000 |
|  | 41-50 years | 0.004 | 0.004 | 1.000 |
|  | 51-60 years | -0.003 | 0.003 | 1.000 |
|  | 61-70 years | -0.006 | 0.003 | 1.000 |
|  | 71-80 years | -0.005 | 0.003 | 1.000 |
|  | 81-90 years | -0.004 | 0.005 | 1.000 |
| 41-50 years | 21-30 years | -0.009 | 0.004 | 0.236 |
|  | 31-40 years | -0.004 | 0.004 | 1.000 |
|  | 51-60 years | -0.007 | 0.003 | 0.438 |
|  | 61-70 years | -0.010 | 0.003 | *0.041* |
|  | 71-80 years | -0.009 | 0.003 | 0.133 |
|  | 81-90 years | -0.008 | 0.005 | 1.000 |
| 51-60 years | 21-30 years | -0.002 | 0.002 | 1.000 |
|  | 31-40 years | 0.003 | 0.003 | 1.000 |
|  | 41-50 years | 0.007 | 0.003 | 0.438 |
|  | 61-70 years | -0.003 | 0.001 | 1.000 |
|  | 71-80 years | -0.002 | 0.002 | 1.000 |
|  | 81-90 years | -0.001 | 0.004 | 1.000 |
| 61-70 years | 21-30 years | 0.000 | 0.002 | 1.000 |
|  | 31-40 years | 0.006 | 0.003 | 1.000 |
|  | 41-50 years | 0.010 | 0.003 | *0.041* |
|  | 51-60 years | 0.003 | 0.001 | 1.000 |
|  | 71-80 years | 0.001 | 0.002 | 1.000 |
|  | 81-90 years | 0.001 | 0.004 | 1.000 |
| 71-80 years | 21-30 years | 0.000 | 0.003 | 1.000 |
|  | 31-40 years | 0.005 | 0.003 | 1.000 |
|  | 41-50 years | 0.009 | 0.003 | 0.133 |
|  | 51-60 years | 0.002 | 0.002 | 1.000 |
|  | 61-70 years | -0.001 | 0.002 | 1.000 |
|  | 81-90 years | 0.001 | 0.004 | 1.000 |
| 81-90 years | 21-30 years | -0.001 | 0.005 | 1.000 |
|  | 31-40 years | 0.004 | 0.005 | 1.000 |
|  | 41-50 years | 0.008 | 0.005 | 1.000 |
|  | 51-60 years | 0.001 | 0.004 | 1.000 |
|  | 61-70 years | -0.001 | 0.004 | 1.000 |
|  | 71-80 years | -0.001 | 0.004 | 1.000 |
| SM | Comparison | Mean difference | Standard error | P-value |
| 21-30 years | 31-40 years | 0.002 | 0.006 | 1.000 |
|  | 41-50 years | 0.009 | 0.006 | 1.000 |
|  | 51-60 years | 0.012 | 0.004 | *0.048* |
|  | 61-70 years | 0.026 | 0.004 | *0.000* |
|  | 71-80 years | 0.046 | 0.004 | *0.000* |
|  | 81-90 years | 0.084 | 0.007 | *0.000* |
| 31-40 years | 21-30 years | -0.002 | 0.006 | 1.000 |
|  | 41-50 years | 0.007 | 0.007 | 1.000 |
|  | 51-60 years | 0.010 | 0.005 | 0.924 |
|  | 61-70 years | 0.024 | 0.005 | *0.000* |
|  | 71-80 years | 0.044 | 0.005 | *0.000* |
|  | 81-90 years | 0.082 | 0.008 | *0.000* |
| 41-50 years | 21-30 years | -0.009 | 0.006 | 1.000 |
|  | 31-40 years | -0.007 | 0.007 | 1.000 |
|  | 51-60 years | 0.003 | 0.005 | 1.000 |
|  | 61-70 years | 0.017 | 0.005 | *0.021* |
|  | 71-80 years | 0.037 | 0.005 | *0.000* |
|  | 81-90 years | 0.075 | 0.008 | *0.000* |
| 51-60 years | 21-30 years | -0.012 | 0.004 | *0.048* |
|  | 31-40 years | -0.010 | 0.005 | 0.924 |
|  | 41-50 years | -0.003 | 0.005 | 1.000 |
|  | 61-70 years | 0.014 | 0.002 | *0.000* |
|  | 71-80 years | 0.033 | 0.003 | *0.000* |
|  | 81-90 years | 0.072 | 0.007 | *0.000* |
| 61-70 years | 21-30 years | -0.026 | 0.004 | *0.000* |
|  | 31-40 years | -0.024 | 0.005 | *0.000* |
|  | 41-50 years | -0.017 | 0.005 | *0.021* |
|  | 51-60 years | -0.014 | 0.002 | *0.000* |
|  | 71-80 years | 0.019 | 0.003 | *0.000* |
|  | 81-90 years | 0.058 | 0.007 | *0.000* |
| 71-80 years | 21-30 years | -0.046 | 0.004 | *0.000* |
|  | 31-40 years | -0.044 | 0.005 | *0.000* |
|  | 41-50 years | -0.037 | 0.005 | *0.000* |
|  | 51-60 years | -0.033 | 0.003 | *0.000* |
|  | 61-70 years | -0.019 | 0.003 | *0.000* |
|  | 81-90 years | 0.038 | 0.007 | *0.000* |
| 81-90 years | 21-30 years | -0.084 | 0.007 | *0.000* |
|  | 31-40 years | -0.082 | 0.008 | *0.000* |
|  | 41-50 years | -0.075 | 0.008 | *0.000* |
|  | 51-60 years | -0.072 | 0.007 | *0.000* |
|  | 61-70 years | -0.058 | 0.007 | *0.000* |
|  | 71-80 years | -0.038 | 0.007 | *0.000* |
| DA | Comparison | Mean difference | Standard error | P-value |
| 21-30 years | 31-40 years | -0.004 | 0.004 | 1.000 |
|  | 41-50 years | -0.002 | 0.004 | 1.000 |
|  | 51-60 years | -0.004 | 0.003 | 1.000 |
|  | 61-70 years | -0.006 | 0.003 | 0.430 |
|  | 71-80 years | 0.004 | 0.003 | 1.000 |
|  | 81-90 years | 0.021 | 0.005 | *0.000* |
| 31-40 years | 21-30 years | 0.004 | 0.004 | 1.000 |
|  | 41-50 years | 0.001 | 0.004 | 1.000 |
|  | 51-60 years | 0.000 | 0.003 | 1.000 |
|  | 61-70 years | -0.002 | 0.003 | 1.000 |
|  | 71-80 years | 0.007 | 0.003 | 0.644 |
|  | 81-90 years | 0.025 | 0.005 | *0.000* |
| 41-50 years | 21-30 years | 0.002 | 0.004 | 1.000 |
|  | 31-40 years | -0.001 | 0.004 | 1.000 |
|  | 51-60 years | -0.001 | 0.003 | 1.000 |
|  | 61-70 years | -0.004 | 0.003 | 1.000 |
|  | 71-80 years | 0.006 | 0.003 | 1.000 |
|  | 81-90 years | 0.023 | 0.005 | *0.000* |
| 51-60 years | 21-30 years | 0.004 | 0.003 | 1.000 |
|  | 31-40 years | 0.000 | 0.003 | 1.000 |
|  | 41-50 years | 0.001 | 0.003 | 1.000 |
|  | 61-70 years | -0.002 | 0.001 | 1.000 |
|  | 71-80 years | 0.007 | 0.002 | *0.001* |
|  | 81-90 years | 0.025 | 0.004 | *0.000* |
| 61-70 years | 21-30 years | 0.006 | 0.003 | 0.430 |
|  | 31-40 years | 0.002 | 0.003 | 1.000 |
|  | 41-50 years | 0.004 | 0.003 | 1.000 |
|  | 51-60 years | 0.002 | 0.001 | 1.000 |
|  | 71-80 years | 0.010 | 0.002 | *0.000* |
|  | 81-90 years | 0.027 | 0.004 | *0.000* |
| 71-80 years | 21-30 years | -0.004 | 0.003 | 1.000 |
|  | 31-40 years | -0.007 | 0.003 | 0.644 |
|  | 41-50 years | -0.006 | 0.003 | 1.000 |
|  | 51-60 years | -0.007 | 0.002 | *0.001* |
|  | 61-70 years | -0.010 | 0.002 | *0.000* |
|  | 81-90 years | 0.017 | 0.004 | *0.001* |
| 81-90 years | 21-30 years | -0.021 | 0.005 | *0.000* |
|  | 31-40 years | -0.025 | 0.005 | *0.000* |
|  | 41-50 years | -0.023 | 0.005 | *0.000* |
|  | 51-60 years | -0.025 | 0.004 | *0.000* |
|  | 61-70 years | -0.027 | 0.004 | *0.000* |
|  | 71-80 years | -0.017 | 0.004 | *0.001* |
| VA | Comparison | Mean difference | Standard error | P-value |
| 21-30 years | 31-40 years | 0.003 | 0.003 | 1.000 |
|  | 41-50 years | 0.001 | 0.003 | 1.000 |
|  | 51-60 years | 0.007 | 0.002 | *0.000* |
|  | 61-70 years | 0.015 | 0.002 | *0.000* |
|  | 71-80 years | 0.021 | 0.002 | *0.000* |
|  | 81-90 years | 0.028 | 0.003 | *0.000* |
| 31-40 years | 21-30 years | -0.003 | 0.003 | 1.000 |
|  | 41-50 years | -0.002 | 0.003 | 1.000 |
|  | 51-60 years | 0.005 | 0.002 | 0.737 |
|  | 61-70 years | 0.012 | 0.002 | *0.000* |
|  | 71-80 years | 0.019 | 0.002 | *0.000* |
|  | 81-90 years | 0.025 | 0.003 | *0.000* |
| 41-50 years | 21-30 years | -0.001 | 0.003 | 1.000 |
|  | 31-40 years | 0.002 | 0.003 | 1.000 |
|  | 51-60 years | 0.006 | 0.002 | 0.083 |
|  | 61-70 years | 0.013 | 0.002 | *0.000* |
|  | 71-80 years | 0.020 | 0.002 | *0.000* |
|  | 81-90 years | 0.027 | 0.003 | *0.000* |
| 51-60 years | 21-30 years | -0.007 | 0.002 | *0.000* |
|  | 31-40 years | -0.005 | 0.002 | 0.737 |
|  | 41-50 years | -0.006 | 0.002 | 0.083 |
|  | 61-70 years | 0.007 | 0.001 | *0.000* |
|  | 71-80 years | 0.014 | 0.001 | *0.000* |
|  | 81-90 years | 0.021 | 0.003 | *0.000* |
| 61-70 years | 21-30 years | -0.015 | 0.002 | *0.000* |
|  | 31-40 years | -0.012 | 0.002 | *0.000* |
|  | 41-50 years | -0.013 | 0.002 | *0.000* |
|  | 51-60 years | -0.007 | 0.001 | *0.000* |
|  | 71-80 years | 0.007 | 0.001 | *0.000* |
|  | 81-90 years | 0.013 | 0.003 | *0.000* |
| 71-80 years | 21-30 years | -0.021 | 0.002 | *0.000* |
|  | 31-40 years | -0.019 | 0.002 | *0.000* |
|  | 41-50 years | -0.020 | 0.002 | *0.000* |
|  | 51-60 years | -0.014 | 0.001 | *0.000* |
|  | 61-70 years | -0.007 | 0.001 | *0.000* |
|  | 81-90 years | 0.007 | 0.003 | 0.495 |
| 81-90 years | 21-30 years | -0.028 | 0.003 | *0.000* |
|  | 31-40 years | -0.025 | 0.003 | *0.000* |
|  | 41-50 years | -0.027 | 0.003 | *0.000* |
|  | 51-60 years | -0.021 | 0.003 | *0.000* |
|  | 61-70 years | -0.013 | 0.003 | *0.000* |
|  | 71-80 years | -0.007 | 0.003 | 0.495 |
| LN | Comparison | Mean difference | Standard error | P-value |
| 21-30 years | 31-40 years | 0.000 | 0.002 | 1.000 |
|  | 41-50 years | 0.000 | 0.002 | 1.000 |
|  | 51-60 years | -0.002 | 0.001 | 0.656 |
|  | 61-70 years | -0.002 | 0.001 | 1.000 |
|  | 71-80 years | -0.003 | 0.001 | 0.486 |
|  | 81-90 years | -0.003 | 0.002 | 1.000 |
| 31-40 years | 21-30 years | 0.000 | 0.002 | 1.000 |
|  | 41-50 years | 0.000 | 0.002 | 1.000 |
|  | 51-60 years | -0.002 | 0.001 | 1.000 |
|  | 61-70 years | -0.002 | 0.001 | 1.000 |
|  | 71-80 years | -0.002 | 0.001 | 1.000 |
|  | 81-90 years | -0.003 | 0.002 | 1.000 |
| 41-50 years | 21-30 years | 0.000 | 0.002 | 1.000 |
|  | 31-40 years | 0.000 | 0.002 | 1.000 |
|  | 51-60 years | -0.002 | 0.001 | 1.000 |
|  | 61-70 years | -0.002 | 0.001 | 1.000 |
|  | 71-80 years | -0.003 | 0.001 | 1.000 |
|  | 81-90 years | -0.003 | 0.002 | 1.000 |
| 51-60 years | 21-30 years | 0.002 | 0.001 | 0.656 |
|  | 31-40 years | 0.002 | 0.001 | 1.000 |
|  | 41-50 years | 0.002 | 0.001 | 1.000 |
|  | 61-70 years | 0.000 | 0.001 | 1.000 |
|  | 71-80 years | 0.000 | 0.001 | 1.000 |
|  | 81-90 years | -0.001 | 0.002 | 1.000 |
| 61-70 years | 21-30 years | 0.002 | 0.001 | 1.000 |
|  | 31-40 years | 0.002 | 0.001 | 1.000 |
|  | 41-50 years | 0.002 | 0.001 | 1.000 |
|  | 51-60 years | 0.000 | 0.001 | 1.000 |
|  | 71-80 years | -0.001 | 0.001 | 1.000 |
|  | 81-90 years | -0.001 | 0.002 | 1.000 |
| 71-80 years | 21-30 years | 0.003 | 0.001 | 0.486 |
|  | 31-40 years | 0.002 | 0.001 | 1.000 |
|  | 41-50 years | 0.003 | 0.001 | 1.000 |
|  | 51-60 years | 0.000 | 0.001 | 1.000 |
|  | 61-70 years | 0.001 | 0.001 | 1.000 |
|  | 81-90 years | 0.000 | 0.002 | 1.000 |
| 81-90 years | 21-30 years | 0.003 | 0.002 | 1.000 |
|  | 31-40 years | 0.003 | 0.002 | 1.000 |
|  | 41-50 years | 0.003 | 0.002 | 1.000 |
|  | 51-60 years | 0.001 | 0.002 | 1.000 |
|  | 61-70 years | 0.001 | 0.002 | 1.000 |
|  | 71-80 years | 0.000 | 0.002 | 1.000 |
| FPN | Comparison | Mean difference | Standard error | P-value |
| 21-30 years | 31-40 years | 0.007 | 0.005 | 1.000 |
|  | 41-50 years | 0.009 | 0.005 | 1.000 |
|  | 51-60 years | 0.008 | 0.003 | 0.278 |
|  | 61-70 years | 0.018 | 0.003 | *0.000* |
|  | 71-80 years | 0.034 | 0.003 | *0.000* |
|  | 81-90 years | 0.050 | 0.006 | *0.000* |
| 31-40 years | 21-30 years | -0.007 | 0.005 | 1.000 |
|  | 41-50 years | 0.003 | 0.005 | 1.000 |
|  | 51-60 years | 0.001 | 0.004 | 1.000 |
|  | 61-70 years | 0.012 | 0.004 | 0.080 |
|  | 71-80 years | 0.027 | 0.004 | *0.000* |
|  | 81-90 years | 0.044 | 0.006 | *0.000* |
| 41-50 years | 21-30 years | -0.009 | 0.005 | 1.000 |
|  | 31-40 years | -0.003 | 0.005 | 1.000 |
|  | 51-60 years | -0.002 | 0.004 | 1.000 |
|  | 61-70 years | 0.009 | 0.004 | 0.458 |
|  | 71-80 years | 0.025 | 0.004 | *0.000* |
|  | 81-90 years | 0.041 | 0.006 | *0.000* |
| 51-60 years | 21-30 years | -0.008 | 0.003 | 0.278 |
|  | 31-40 years | -0.001 | 0.004 | 1.000 |
|  | 41-50 years | 0.002 | 0.004 | 1.000 |
|  | 61-70 years | 0.011 | 0.002 | *0.000* |
|  | 71-80 years | 0.026 | 0.002 | *0.000* |
|  | 81-90 years | 0.043 | 0.005 | *0.000* |
| 61-70 years | 21-30 years | -0.018 | 0.003 | *0.000* |
|  | 31-40 years | -0.012 | 0.004 | 0.080 |
|  | 41-50 years | -0.009 | 0.004 | 0.458 |
|  | 51-60 years | -0.011 | 0.002 | *0.000* |
|  | 71-80 years | 0.016 | 0.002 | *0.000* |
|  | 81-90 years | 0.032 | 0.005 | *0.000* |
| 71-80 years | 21-30 years | -0.034 | 0.003 | *0.000* |
|  | 31-40 years | -0.027 | 0.004 | *0.000* |
|  | 41-50 years | -0.025 | 0.004 | *0.000* |
|  | 51-60 years | -0.026 | 0.002 | *0.000* |
|  | 61-70 years | -0.016 | 0.002 | *0.000* |
|  | 81-90 years | 0.016 | 0.005 | *0.035* |
| 81-90 years | 21-30 years | -0.050 | 0.006 | *0.000* |
|  | 31-40 years | -0.044 | 0.006 | *0.000* |
|  | 41-50 years | -0.041 | 0.006 | *0.000* |
|  | 51-60 years | -0.043 | 0.005 | *0.000* |
|  | 61-70 years | -0.032 | 0.005 | *0.000* |
|  | 71-80 years | -0.016 | 0.005 | *0.035* |
| DMN | Comparison | Mean difference | Standard error | P-value |
| 21-30 years | 31-40 years | 0.000 | 0.008 | 1.000 |
|  | 41-50 years | -0.005 | 0.008 | 1.000 |
|  | 51-60 years | 0.005 | 0.005 | 1.000 |
|  | 61-70 years | 0.009 | 0.005 | 1.000 |
|  | 71-80 years | 0.030 | 0.005 | *0.000* |
|  | 81-90 years | 0.051 | 0.009 | *0.000* |
| 31-40 years | 21-30 years | 0.000 | 0.008 | 1.000 |
|  | 41-50 years | -0.004 | 0.009 | 1.000 |
|  | 51-60 years | 0.005 | 0.006 | 1.000 |
|  | 61-70 years | 0.009 | 0.006 | 1.000 |
|  | 71-80 years | 0.030 | 0.007 | *0.000* |
|  | 81-90 years | 0.051 | 0.010 | *0.000* |
| 41-50 years | 21-30 years | 0.005 | 0.008 | 1.000 |
|  | 31-40 years | 0.004 | 0.009 | 1.000 |
|  | 51-60 years | 0.010 | 0.006 | 1.000 |
|  | 61-70 years | 0.013 | 0.006 | 0.829 |
|  | 71-80 years | 0.034 | 0.007 | *0.000* |
|  | 81-90 years | 0.056 | 0.010 | *0.000* |
| 51-60 years | 21-30 years | -0.005 | 0.005 | 1.000 |
|  | 31-40 years | -0.005 | 0.006 | 1.000 |
|  | 41-50 years | -0.010 | 0.006 | 1.000 |
|  | 61-70 years | 0.003 | 0.003 | 1.000 |
|  | 71-80 years | 0.024 | 0.003 | *0.000* |
|  | 81-90 years | 0.046 | 0.008 | *0.000* |
| 61-70 years | 21-30 years | -0.009 | 0.005 | 1.000 |
|  | 31-40 years | -0.009 | 0.006 | 1.000 |
|  | 41-50 years | -0.013 | 0.006 | 0.829 |
|  | 51-60 years | -0.003 | 0.003 | 1.000 |
|  | 71-80 years | 0.021 | 0.004 | *0.000* |
|  | 81-90 years | 0.042 | 0.008 | *0.000* |
| 71-80 years | 21-30 years | -0.030 | 0.005 | *0.000* |
|  | 31-40 years | -0.030 | 0.007 | *0.000* |
|  | 41-50 years | -0.034 | 0.007 | *0.000* |
|  | 51-60 years | -0.024 | 0.003 | *0.000* |
|  | 61-70 years | -0.021 | 0.004 | *0.000* |
|  | 81-90 years | 0.022 | 0.008 | 0.230 |
| 81-90 years | 21-30 years | -0.051 | 0.009 | *0.000* |
|  | 31-40 years | -0.051 | 0.010 | *0.000* |
|  | 41-50 years | -0.056 | 0.010 | *0.000* |
|  | 51-60 years | -0.046 | 0.008 | *0.000* |
|  | 61-70 years | -0.042 | 0.008 | *0.000* |
|  | 71-80 years | -0.022 | 0.008 | 0.230 |

VIS, CC subregion connecting to the visual network; SM, CC subregion connecting to the somatomotor network; DA, CC subregion connecting to the dorsal attention network; VA, CC subregion connecting to the ventral attention network; LN, CC subregion connecting to the limbic network; FPN, CC subregion connecting to the frontoparietal network; DMN, CC subregion connecting to the default mode network.

**Supplementary Table 3. The comparison of connection probability between age groups for 17 CC subregions with Bonferroni-corrected post hoc tests**

| Subregion 1 | Comparison | Mean difference | Standard error | P-value |
| --- | --- | --- | --- | --- |
| 21-30 years | 31-40 years | 0.002 | 0.002 | 1.000 |
|  | 41-50 years | 0.003 | 0.002 | 0.682 |
|  | 51-60 years | 0.000 | 0.001 | 1.000 |
|  | 61-70 years | 0.001 | 0.001 | 1.000 |
|  | 71-80 years | 0.001 | 0.001 | 1.000 |
|  | 81-90 years | 0.004 | 0.002 | 1.000 |
| 31-40 years | 21-30 years | -0.002 | 0.002 | 1.000 |
|  | 41-50 years | 0.002 | 0.002 | 1.000 |
|  | 51-60 years | -0.001 | 0.001 | 1.000 |
|  | 61-70 years | -0.001 | 0.001 | 1.000 |
|  | 71-80 years | 0.000 | 0.001 | 1.000 |
|  | 81-90 years | 0.002 | 0.002 | 1.000 |
| 41-50 years | 21-30 years | -0.003 | 0.002 | 0.682 |
|  | 31-40 years | -0.002 | 0.002 | 1.000 |
|  | 51-60 years | -0.003 | 0.001 | 0.599 |
|  | 61-70 years | -0.003 | 0.001 | 0.991 |
|  | 71-80 years | -0.002 | 0.001 | 1.000 |
|  | 81-90 years | 0.000 | 0.002 | 1.000 |
| 51-60 years | 21-30 years | 0.000 | 0.001 | 1.000 |
|  | 31-40 years | 0.001 | 0.001 | 1.000 |
|  | 41-50 years | 0.003 | 0.001 | 0.599 |
|  | 61-70 years | 0.000 | 0.001 | 1.000 |
|  | 71-80 years | 0.001 | 0.001 | 1.000 |
|  | 81-90 years | 0.003 | 0.002 | 1.000 |
| 61-70 years | 21-30 years | -0.001 | 0.001 | 1.000 |
|  | 31-40 years | 0.001 | 0.001 | 1.000 |
|  | 41-50 years | 0.003 | 0.001 | 0.991 |
|  | 51-60 years | 0.000 | 0.001 | 1.000 |
|  | 71-80 years | 0.001 | 0.001 | 1.000 |
|  | 81-90 years | 0.003 | 0.002 | 1.000 |
| 71-80 years | 21-30 years | -0.001 | 0.001 | 1.000 |
|  | 31-40 years | 0.000 | 0.001 | 1.000 |
|  | 41-50 years | 0.002 | 0.001 | 1.000 |
|  | 51-60 years | -0.001 | 0.001 | 1.000 |
|  | 61-70 years | -0.001 | 0.001 | 1.000 |
|  | 81-90 years | 0.002 | 0.002 | 1.000 |
| 81-90 years | 21-30 years | -0.004 | 0.002 | 1.000 |
|  | 31-40 years | -0.002 | 0.002 | 1.000 |
|  | 41-50 years | 0.000 | 0.002 | 1.000 |
|  | 51-60 years | -0.003 | 0.002 | 1.000 |
|  | 61-70 years | -0.003 | 0.002 | 1.000 |
|  | 71-80 years | -0.002 | 0.002 | 1.000 |
| Subregion 2 | Comparison | Mean difference | Standard error | P-value |
| 21-30 years | 31-40 years | 0.003 | 0.002 | 1.000 |
|  | 41-50 years | 0.004 | 0.002 | 1.000 |
|  | 51-60 years | 0.002 | 0.002 | 1.000 |
|  | 61-70 years | 0.001 | 0.002 | 1.000 |
|  | 71-80 years | 0.002 | 0.002 | 1.000 |
|  | 81-90 years | 0.003 | 0.003 | 1.000 |
| 31-40 years | 21-30 years | -0.003 | 0.002 | 1.000 |
|  | 41-50 years | 0.001 | 0.003 | 1.000 |
|  | 51-60 years | -0.001 | 0.002 | 1.000 |
|  | 61-70 years | -0.001 | 0.002 | 1.000 |
|  | 71-80 years | -0.001 | 0.002 | 1.000 |
|  | 81-90 years | 0.001 | 0.003 | 1.000 |
| 41-50 years | 21-30 years | -0.004 | 0.002 | 1.000 |
|  | 31-40 years | -0.001 | 0.003 | 1.000 |
|  | 51-60 years | -0.002 | 0.002 | 1.000 |
|  | 61-70 years | -0.002 | 0.002 | 1.000 |
|  | 71-80 years | -0.002 | 0.002 | 1.000 |
|  | 81-90 years | 0.000 | 0.003 | 1.000 |
| 51-60 years | 21-30 years | -0.002 | 0.002 | 1.000 |
|  | 31-40 years | 0.001 | 0.002 | 1.000 |
|  | 41-50 years | 0.002 | 0.002 | 1.000 |
|  | 61-70 years | -0.001 | 0.001 | 1.000 |
|  | 71-80 years | 0.000 | 0.001 | 1.000 |
|  | 81-90 years | 0.001 | 0.003 | 1.000 |
| 61-70 years | 21-30 years | -0.001 | 0.002 | 1.000 |
|  | 31-40 years | 0.001 | 0.002 | 1.000 |
|  | 41-50 years | 0.002 | 0.002 | 1.000 |
|  | 51-60 years | 0.001 | 0.001 | 1.000 |
|  | 71-80 years | 0.001 | 0.001 | 1.000 |
|  | 81-90 years | 0.002 | 0.003 | 1.000 |
| 71-80 years | 21-30 years | -0.002 | 0.002 | 1.000 |
|  | 31-40 years | 0.001 | 0.002 | 1.000 |
|  | 41-50 years | 0.002 | 0.002 | 1.000 |
|  | 51-60 years | 0.000 | 0.001 | 1.000 |
|  | 61-70 years | -0.001 | 0.001 | 1.000 |
|  | 81-90 years | 0.001 | 0.003 | 1.000 |
| 81-90 years | 21-30 years | -0.003 | 0.003 | 1.000 |
|  | 31-40 years | -0.001 | 0.003 | 1.000 |
|  | 41-50 years | 0.000 | 0.003 | 1.000 |
|  | 51-60 years | -0.001 | 0.003 | 1.000 |
|  | 61-70 years | -0.002 | 0.003 | 1.000 |
|  | 71-80 years | -0.001 | 0.003 | 1.000 |
| Subregion 3 | Comparison | Mean difference | Standard error | P-value |
| 21-30 years | 31-40 years | 0.002 | 0.006 | 1.000 |
|  | 41-50 years | 0.008 | 0.006 | 1.000 |
|  | 51-60 years | 0.012 | 0.004 | *0.016* |
|  | 61-70 years | 0.026 | 0.004 | *0.000* |
|  | 71-80 years | 0.045 | 0.004 | *0.000* |
|  | 81-90 years | 0.079 | 0.007 | *0.000* |
| 31-40 years | 21-30 years | -0.002 | 0.006 | 1.000 |
|  | 41-50 years | 0.006 | 0.006 | 1.000 |
|  | 51-60 years | 0.010 | 0.005 | 0.649 |
|  | 61-70 years | 0.024 | 0.005 | *0.000* |
|  | 71-80 years | 0.043 | 0.005 | *0.000* |
|  | 81-90 years | 0.076 | 0.007 | *0.000* |
| 41-50 years | 21-30 years | -0.008 | 0.006 | 1.000 |
|  | 31-40 years | -0.006 | 0.006 | 1.000 |
|  | 51-60 years | 0.005 | 0.005 | 1.000 |
|  | 61-70 years | 0.018 | 0.005 | *0.003* |
|  | 71-80 years | 0.037 | 0.005 | *0.000* |
|  | 81-90 years | 0.071 | 0.007 | *0.000* |
| 51-60 years | 21-30 years | -0.012 | 0.004 | *0.016* |
|  | 31-40 years | -0.010 | 0.005 | 0.649 |
|  | 41-50 years | -0.005 | 0.005 | 1.000 |
|  | 61-70 years | 0.014 | 0.002 | *0.000* |
|  | 71-80 years | 0.032 | 0.003 | *0.000* |
|  | 81-90 years | 0.066 | 0.006 | *0.000* |
| 61-70 years | 21-30 years | -0.026 | 0.004 | *0.000* |
|  | 31-40 years | -0.024 | 0.005 | *0.000* |
|  | 41-50 years | -0.018 | 0.005 | *0.003* |
|  | 51-60 years | -0.014 | 0.002 | *0.000* |
|  | 71-80 years | 0.019 | 0.003 | *0.000* |
|  | 81-90 years | 0.053 | 0.006 | *0.000* |
| 71-80 years | 21-30 years | -0.045 | 0.004 | *0.000* |
|  | 31-40 years | -0.043 | 0.005 | *0.000* |
|  | 41-50 years | -0.037 | 0.005 | *0.000* |
|  | 51-60 years | -0.032 | 0.003 | *0.000* |
|  | 61-70 years | -0.019 | 0.003 | *0.000* |
|  | 81-90 years | 0.034 | 0.006 | *0.000* |
| 81-90 years | 21-30 years | -0.079 | 0.007 | *0.000* |
|  | 31-40 years | -0.076 | 0.007 | *0.000* |
|  | 41-50 years | -0.071 | 0.007 | *0.000* |
|  | 51-60 years | -0.066 | 0.006 | *0.000* |
|  | 61-70 years | -0.053 | 0.006 | *0.000* |
|  | 71-80 years | -0.034 | 0.006 | *0.000* |
| Subregion 4 | Comparison | Mean difference | Standard error | P-value |
| 21-30 years | 31-40 years | 0.000 | 0.001 | 1.000 |
|  | 41-50 years | 0.000 | 0.001 | 1.000 |
|  | 51-60 years | 0.000 | 0.001 | 1.000 |
|  | 61-70 years | 0.000 | 0.001 | 1.000 |
|  | 71-80 years | 0.000 | 0.001 | 1.000 |
|  | 81-90 years | 0.002 | 0.001 | 1.000 |
| 31-40 years | 21-30 years | 0.000 | 0.001 | 1.000 |
|  | 41-50 years | 0.000 | 0.001 | 1.000 |
|  | 51-60 years | -0.001 | 0.001 | 1.000 |
|  | 61-70 years | 0.000 | 0.001 | 1.000 |
|  | 71-80 years | -0.001 | 0.001 | 1.000 |
|  | 81-90 years | 0.002 | 0.002 | 1.000 |
| 41-50 years | 21-30 years | 0.000 | 0.001 | 1.000 |
|  | 31-40 years | 0.000 | 0.001 | 1.000 |
|  | 51-60 years | -0.001 | 0.001 | 1.000 |
|  | 61-70 years | -0.001 | 0.001 | 1.000 |
|  | 71-80 years | -0.001 | 0.001 | 1.000 |
|  | 81-90 years | 0.001 | 0.002 | 1.000 |
| 51-60 years | 21-30 years | 0.000 | 0.001 | 1.000 |
|  | 31-40 years | 0.001 | 0.001 | 1.000 |
|  | 41-50 years | 0.001 | 0.001 | 1.000 |
|  | 61-70 years | 0.000 | 0.000 | 1.000 |
|  | 71-80 years | 0.000 | 0.001 | 1.000 |
|  | 81-90 years | 0.002 | 0.001 | 1.000 |
| 61-70 years | 21-30 years | 0.000 | 0.001 | 1.000 |
|  | 31-40 years | 0.000 | 0.001 | 1.000 |
|  | 41-50 years | 0.001 | 0.001 | 1.000 |
|  | 51-60 years | 0.000 | 0.000 | 1.000 |
|  | 71-80 years | 0.000 | 0.001 | 1.000 |
|  | 81-90 years | 0.002 | 0.001 | 1.000 |
| 71-80 years | 21-30 years | 0.000 | 0.001 | 1.000 |
|  | 31-40 years | 0.001 | 0.001 | 1.000 |
|  | 41-50 years | 0.001 | 0.001 | 1.000 |
|  | 51-60 years | 0.000 | 0.001 | 1.000 |
|  | 61-70 years | 0.000 | 0.001 | 1.000 |
|  | 81-90 years | 0.002 | 0.001 | 1.000 |
| 81-90 years | 21-30 years | -0.002 | 0.001 | 1.000 |
|  | 31-40 years | -0.002 | 0.002 | 1.000 |
|  | 41-50 years | -0.001 | 0.002 | 1.000 |
|  | 51-60 years | -0.002 | 0.001 | 1.000 |
|  | 61-70 years | -0.002 | 0.001 | 1.000 |
|  | 71-80 years | -0.002 | 0.001 | 1.000 |
| Subregion 5 | Comparison | Mean difference | Standard error | P-value |
| 21-30 years | 31-40 years | -0.001 | 0.003 | 1.000 |
|  | 41-50 years | -0.003 | 0.003 | 1.000 |
|  | 51-60 years | -0.003 | 0.002 | 1.000 |
|  | 61-70 years | -0.008 | 0.002 | *0.002* |
|  | 71-80 years | -0.003 | 0.002 | 1.000 |
|  | 81-90 years | 0.008 | 0.004 | 0.831 |
| 31-40 years | 21-30 years | 0.001 | 0.003 | 1.000 |
|  | 41-50 years | -0.002 | 0.003 | 1.000 |
|  | 51-60 years | -0.002 | 0.003 | 1.000 |
|  | 61-70 years | -0.007 | 0.003 | 0.154 |
|  | 71-80 years | -0.002 | 0.003 | 1.000 |
|  | 81-90 years | 0.009 | 0.004 | 0.641 |
| 41-50 years | 21-30 years | 0.003 | 0.003 | 1.000 |
|  | 31-40 years | 0.002 | 0.003 | 1.000 |
|  | 51-60 years | 0.000 | 0.003 | 1.000 |
|  | 61-70 years | -0.005 | 0.003 | 0.921 |
|  | 71-80 years | 0.000 | 0.003 | 1.000 |
|  | 81-90 years | 0.011 | 0.004 | 0.185 |
| 51-60 years | 21-30 years | 0.003 | 0.002 | 1.000 |
|  | 31-40 years | 0.002 | 0.003 | 1.000 |
|  | 41-50 years | 0.000 | 0.003 | 1.000 |
|  | 61-70 years | -0.005 | 0.001 | *0.000* |
|  | 71-80 years | 0.000 | 0.001 | 1.000 |
|  | 81-90 years | 0.011 | 0.003 | *0.025* |
| 61-70 years | 21-30 years | 0.008 | 0.002 | *0.002* |
|  | 31-40 years | 0.007 | 0.003 | 0.154 |
|  | 41-50 years | 0.005 | 0.003 | 0.921 |
|  | 51-60 years | 0.005 | 0.001 | *0.000* |
|  | 71-80 years | 0.005 | 0.001 | *0.010* |
|  | 81-90 years | 0.016 | 0.003 | *0.000* |
| 71-80 years | 21-30 years | 0.003 | 0.002 | 1.000 |
|  | 31-40 years | 0.002 | 0.003 | 1.000 |
|  | 41-50 years | 0.000 | 0.003 | 1.000 |
|  | 51-60 years | 0.000 | 0.001 | 1.000 |
|  | 61-70 years | -0.005 | 0.001 | *0.010* |
|  | 81-90 years | 0.011 | 0.003 | *0.038* |
| 81-90 years | 21-30 years | -0.008 | 0.004 | 0.831 |
|  | 31-40 years | -0.009 | 0.004 | 0.641 |
|  | 41-50 years | -0.011 | 0.004 | 0.185 |
|  | 51-60 years | -0.011 | 0.003 | *0.025* |
|  | 61-70 years | -0.016 | 0.003 | *0.000* |
|  | 71-80 years | -0.011 | 0.003 | *0.038* |
| Subregion 6 | Comparison | Mean difference | Standard error | P-value |
| 21-30 years | 31-40 years | -0.002 | 0.002 | 1.000 |
|  | 41-50 years | 0.003 | 0.002 | 1.000 |
|  | 51-60 years | 0.001 | 0.001 | 1.000 |
|  | 61-70 years | 0.004 | 0.001 | 0.161 |
|  | 71-80 years | 0.010 | 0.002 | *0.000* |
|  | 81-90 years | 0.017 | 0.003 | *0.000* |
| 31-40 years | 21-30 years | 0.002 | 0.002 | 1.000 |
|  | 41-50 years | 0.006 | 0.002 | 0.530 |
|  | 51-60 years | 0.004 | 0.002 | 0.866 |
|  | 61-70 years | 0.006 | 0.002 | *0.019* |
|  | 71-80 years | 0.012 | 0.002 | *0.000* |
|  | 81-90 years | 0.020 | 0.003 | *0.000* |
| 41-50 years | 21-30 years | -0.003 | 0.002 | 1.000 |
|  | 31-40 years | -0.006 | 0.002 | 0.530 |
|  | 51-60 years | -0.002 | 0.002 | 1.000 |
|  | 61-70 years | 0.001 | 0.002 | 1.000 |
|  | 71-80 years | 0.007 | 0.002 | *0.009* |
|  | 81-90 years | 0.014 | 0.003 | *0.000* |
| 51-60 years | 21-30 years | -0.001 | 0.001 | 1.000 |
|  | 31-40 years | -0.004 | 0.002 | 0.866 |
|  | 41-50 years | 0.002 | 0.002 | 1.000 |
|  | 61-70 years | 0.002 | 0.001 | 0.079 |
|  | 71-80 years | 0.009 | 0.001 | *0.000* |
|  | 81-90 years | 0.016 | 0.002 | *0.000* |
| 61-70 years | 21-30 years | -0.004 | 0.001 | 0.161 |
|  | 31-40 years | -0.006 | 0.002 | *0.019* |
|  | 41-50 years | -0.001 | 0.002 | 1.000 |
|  | 51-60 years | -0.002 | 0.001 | 0.079 |
|  | 71-80 years | 0.006 | 0.001 | *0.000* |
|  | 81-90 years | 0.014 | 0.002 | *0.000* |
| 71-80 years | 21-30 years | -0.010 | 0.002 | *0.000* |
|  | 31-40 years | -0.012 | 0.002 | *0.000* |
|  | 41-50 years | -0.007 | 0.002 | *0.009* |
|  | 51-60 years | -0.009 | 0.001 | *0.000* |
|  | 61-70 years | -0.006 | 0.001 | *0.000* |
|  | 81-90 years | 0.007 | 0.002 | 0.051 |
| 81-90 years | 21-30 years | -0.017 | 0.003 | *0.000* |
|  | 31-40 years | -0.020 | 0.003 | *0.000* |
|  | 41-50 years | -0.014 | 0.003 | *0.000* |
|  | 51-60 years | -0.016 | 0.002 | *0.000* |
|  | 61-70 years | -0.014 | 0.002 | *0.000* |
|  | 71-80 years | -0.007 | 0.002 | 0.051 |
| Subregion 7 | Comparison | Mean difference | Standard error | P-value |
| 21-30 years | 31-40 years | 0.001 | 0.003 | 1.000 |
|  | 41-50 years | 0.000 | 0.002 | 1.000 |
|  | 51-60 years | 0.006 | 0.002 | *0.005* |
|  | 61-70 years | 0.012 | 0.002 | *0.000* |
|  | 71-80 years | 0.017 | 0.002 | *0.000* |
|  | 81-90 years | 0.023 | 0.003 | *0.000* |
| 31-40 years | 21-30 years | -0.001 | 0.003 | 1.000 |
|  | 41-50 years | -0.001 | 0.003 | 1.000 |
|  | 51-60 years | 0.005 | 0.002 | 0.406 |
|  | 61-70 years | 0.011 | 0.002 | *0.000* |
|  | 71-80 years | 0.016 | 0.002 | *0.000* |
|  | 81-90 years | 0.022 | 0.003 | *0.000* |
| 41-50 years | 21-30 years | 0.000 | 0.002 | 1.000 |
|  | 31-40 years | 0.001 | 0.003 | 1.000 |
|  | 51-60 years | 0.006 | 0.002 | 0.092 |
|  | 61-70 years | 0.012 | 0.002 | *0.000* |
|  | 71-80 years | 0.017 | 0.002 | *0.000* |
|  | 81-90 years | 0.023 | 0.003 | *0.000* |
| 51-60 years | 21-30 years | -0.006 | 0.002 | *0.005* |
|  | 31-40 years | -0.005 | 0.002 | 0.406 |
|  | 41-50 years | -0.006 | 0.002 | 0.092 |
|  | 61-70 years | 0.006 | 0.001 | *0.000* |
|  | 71-80 years | 0.011 | 0.001 | *0.000* |
|  | 81-90 years | 0.017 | 0.003 | *0.000* |
| 61-70 years | 21-30 years | -0.012 | 0.002 | *0.000* |
|  | 31-40 years | -0.011 | 0.002 | *0.000* |
|  | 41-50 years | -0.012 | 0.002 | *0.000* |
|  | 51-60 years | -0.006 | 0.001 | *0.000* |
|  | 71-80 years | 0.006 | 0.001 | *0.000* |
|  | 81-90 years | 0.011 | 0.003 | *0.001* |
| 71-80 years | 21-30 years | -0.017 | 0.002 | *0.000* |
|  | 31-40 years | -0.016 | 0.002 | *0.000* |
|  | 41-50 years | -0.017 | 0.002 | *0.000* |
|  | 51-60 years | -0.011 | 0.001 | *0.000* |
|  | 61-70 years | -0.006 | 0.001 | *0.000* |
|  | 81-90 years | 0.006 | 0.003 | 0.792 |
| 81-90 years | 21-30 years | -0.023 | 0.003 | *0.000* |
|  | 31-40 years | -0.022 | 0.003 | *0.000* |
|  | 41-50 years | -0.023 | 0.003 | *0.000* |
|  | 51-60 years | -0.017 | 0.003 | *0.000* |
|  | 61-70 years | -0.011 | 0.003 | *0.001* |
|  | 71-80 years | -0.006 | 0.003 | 0.792 |
| Subregion 8 | Comparison | Mean difference | Standard error | P-value |
| 21-30 years | 31-40 years | 0.005 | 0.002 | 0.732 |
|  | 41-50 years | 0.007 | 0.002 | 0.068 |
|  | 51-60 years | 0.009 | 0.002 | *0.000* |
|  | 61-70 years | 0.014 | 0.002 | *0.000* |
|  | 71-80 years | 0.021 | 0.002 | *0.000* |
|  | 81-90 years | 0.032 | 0.003 | *0.000* |
| 31-40 years | 21-30 years | -0.005 | 0.002 | 0.732 |
|  | 41-50 years | 0.002 | 0.003 | 1.000 |
|  | 51-60 years | 0.004 | 0.002 | 1.000 |
|  | 61-70 years | 0.009 | 0.002 | *0.001* |
|  | 71-80 years | 0.016 | 0.002 | *0.000* |
|  | 81-90 years | 0.027 | 0.003 | *0.000* |
| 41-50 years | 21-30 years | -0.007 | 0.002 | 0.068 |
|  | 31-40 years | -0.002 | 0.003 | 1.000 |
|  | 51-60 years | 0.002 | 0.002 | 1.000 |
|  | 61-70 years | 0.007 | 0.002 | *0.020* |
|  | 71-80 years | 0.014 | 0.002 | *0.000* |
|  | 81-90 years | 0.025 | 0.003 | *0.000* |
| 51-60 years | 21-30 years | -0.009 | 0.002 | *0.000* |
|  | 31-40 years | -0.004 | 0.002 | 1.000 |
|  | 41-50 years | -0.002 | 0.002 | 1.000 |
|  | 61-70 years | 0.005 | 0.001 | *0.000* |
|  | 71-80 years | 0.012 | 0.001 | *0.000* |
|  | 81-90 years | 0.023 | 0.003 | *0.000* |
| 61-70 years | 21-30 years | -0.014 | 0.002 | *0.000* |
|  | 31-40 years | -0.009 | 0.002 | *0.001* |
|  | 41-50 years | -0.007 | 0.002 | *0.020* |
|  | 51-60 years | -0.005 | 0.001 | *0.000* |
|  | 71-80 years | 0.007 | 0.001 | *0.000* |
|  | 81-90 years | 0.018 | 0.003 | *0.000* |
| 71-80 years | 21-30 years | -0.021 | 0.002 | *0.000* |
|  | 31-40 years | -0.016 | 0.002 | *0.000* |
|  | 41-50 years | -0.014 | 0.002 | *0.000* |
|  | 51-60 years | -0.012 | 0.001 | *0.000* |
|  | 61-70 years | -0.007 | 0.001 | *0.000* |
|  | 81-90 years | 0.011 | 0.003 | *0.001* |
| 81-90 years | 21-30 years | -0.032 | 0.003 | *0.000* |
|  | 31-40 years | -0.027 | 0.003 | *0.000* |
|  | 41-50 years | -0.025 | 0.003 | *0.000* |
|  | 51-60 years | -0.023 | 0.003 | *0.000* |
|  | 61-70 years | -0.018 | 0.003 | *0.000* |
|  | 71-80 years | -0.011 | 0.003 | *0.001* |
| Subregion 9 | Comparison | Mean difference | Standard error | P-value |
| 21-30 years | 31-40 years | 0.000 | 0.000 | 1.000 |
|  | 41-50 years | 0.000 | 0.000 | 1.000 |
|  | 51-60 years | 0.000 | 0.000 | 1.000 |
|  | 61-70 years | 0.000 | 0.000 | 1.000 |
|  | 71-80 years | 0.000 | 0.000 | *0.034* |
|  | 81-90 years | 0.000 | 0.000 | 1.000 |
| 31-40 years | 21-30 years | 0.000 | 0.000 | 1.000 |
|  | 41-50 years | 0.000 | 0.000 | 1.000 |
|  | 51-60 years | 0.000 | 0.000 | 1.000 |
|  | 61-70 years | 0.000 | 0.000 | 1.000 |
|  | 71-80 years | 0.000 | 0.000 | 1.000 |
|  | 81-90 years | 0.000 | 0.000 | 1.000 |
| 41-50 years | 21-30 years | 0.000 | 0.000 | 1.000 |
|  | 31-40 years | 0.000 | 0.000 | 1.000 |
|  | 51-60 years | 0.000 | 0.000 | 1.000 |
|  | 61-70 years | 0.000 | 0.000 | 1.000 |
|  | 71-80 years | 0.000 | 0.000 | 1.000 |
|  | 81-90 years | 0.000 | 0.000 | 1.000 |
| 51-60 years | 21-30 years | 0.000 | 0.000 | 1.000 |
|  | 31-40 years | 0.000 | 0.000 | 1.000 |
|  | 41-50 years | 0.000 | 0.000 | 1.000 |
|  | 61-70 years | 0.000 | 0.000 | 1.000 |
|  | 71-80 years | 0.000 | 0.000 | *0.022* |
|  | 81-90 years | 0.000 | 0.000 | 1.000 |
| 61-70 years | 21-30 years | 0.000 | 0.000 | 1.000 |
|  | 31-40 years | 0.000 | 0.000 | 1.000 |
|  | 41-50 years | 0.000 | 0.000 | 1.000 |
|  | 51-60 years | 0.000 | 0.000 | 1.000 |
|  | 71-80 years | 0.000 | 0.000 | 0.119 |
|  | 81-90 years | 0.000 | 0.000 | 1.000 |
| 71-80 years | 21-30 years | 0.000 | 0.000 | *0.034* |
|  | 31-40 years | 0.000 | 0.000 | 1.000 |
|  | 41-50 years | 0.000 | 0.000 | 1.000 |
|  | 51-60 years | 0.000 | 0.000 | *0.022* |
|  | 61-70 years | 0.000 | 0.000 | 0.119 |
|  | 81-90 years | 0.000 | 0.000 | 1.000 |
| 81-90 years | 21-30 years | 0.000 | 0.000 | 1.000 |
|  | 31-40 years | 0.000 | 0.000 | 1.000 |
|  | 41-50 years | 0.000 | 0.000 | 1.000 |
|  | 51-60 years | 0.000 | 0.000 | 1.000 |
|  | 61-70 years | 0.000 | 0.000 | 1.000 |
|  | 71-80 years | 0.000 | 0.000 | 1.000 |
| Subregion 10 | Comparison | Mean difference | Standard error | P-value |
| 21-30 years | 31-40 years | -0.001 | 0.002 | 1.000 |
|  | 41-50 years | 0.000 | 0.002 | 1.000 |
|  | 51-60 years | -0.004 | 0.001 | 0.188 |
|  | 61-70 years | -0.003 | 0.001 | 1.000 |
|  | 71-80 years | -0.003 | 0.002 | 1.000 |
|  | 81-90 years | -0.003 | 0.003 | 1.000 |
| 31-40 years | 21-30 years | 0.001 | 0.002 | 1.000 |
|  | 41-50 years | 0.001 | 0.003 | 1.000 |
|  | 51-60 years | -0.003 | 0.002 | 1.000 |
|  | 61-70 years | -0.002 | 0.002 | 1.000 |
|  | 71-80 years | -0.002 | 0.002 | 1.000 |
|  | 81-90 years | -0.002 | 0.003 | 1.000 |
| 41-50 years | 21-30 years | 0.000 | 0.002 | 1.000 |
|  | 31-40 years | -0.001 | 0.003 | 1.000 |
|  | 51-60 years | -0.004 | 0.002 | 0.877 |
|  | 61-70 years | -0.003 | 0.002 | 1.000 |
|  | 71-80 years | -0.003 | 0.002 | 1.000 |
|  | 81-90 years | -0.003 | 0.003 | 1.000 |
| 51-60 years | 21-30 years | 0.004 | 0.001 | 0.188 |
|  | 31-40 years | 0.003 | 0.002 | 1.000 |
|  | 41-50 years | 0.004 | 0.002 | 0.877 |
|  | 61-70 years | 0.001 | 0.001 | 1.000 |
|  | 71-80 years | 0.001 | 0.001 | 1.000 |
|  | 81-90 years | 0.001 | 0.002 | 1.000 |
| 61-70 years | 21-30 years | 0.003 | 0.001 | 1.000 |
|  | 31-40 years | 0.002 | 0.002 | 1.000 |
|  | 41-50 years | 0.003 | 0.002 | 1.000 |
|  | 51-60 years | -0.001 | 0.001 | 1.000 |
|  | 71-80 years | 0.000 | 0.001 | 1.000 |
|  | 81-90 years | 0.000 | 0.002 | 1.000 |
| 71-80 years | 21-30 years | 0.003 | 0.002 | 1.000 |
|  | 31-40 years | 0.002 | 0.002 | 1.000 |
|  | 41-50 years | 0.003 | 0.002 | 1.000 |
|  | 51-60 years | -0.001 | 0.001 | 1.000 |
|  | 61-70 years | 0.000 | 0.001 | 1.000 |
|  | 81-90 years | 0.001 | 0.002 | 1.000 |
| 81-90 years | 21-30 years | 0.003 | 0.003 | 1.000 |
|  | 31-40 years | 0.002 | 0.003 | 1.000 |
|  | 41-50 years | 0.003 | 0.003 | 1.000 |
|  | 51-60 years | -0.001 | 0.002 | 1.000 |
|  | 61-70 years | 0.000 | 0.002 | 1.000 |
|  | 71-80 years | -0.001 | 0.002 | 1.000 |
| Subregion 11 | Comparison | Mean difference | Standard error | P-value |
| 21-30 years | 31-40 years | 0.002 | 0.003 | 1.000 |
|  | 41-50 years | -0.003 | 0.003 | 1.000 |
|  | 51-60 years | -0.006 | 0.002 | *0.026* |
|  | 61-70 years | -0.010 | 0.002 | *0.000* |
|  | 71-80 years | -0.010 | 0.002 | *0.000* |
|  | 81-90 years | -0.027 | 0.004 | *0.000* |
| 31-40 years | 21-30 years | -0.002 | 0.003 | 1.000 |
|  | 41-50 years | -0.005 | 0.003 | 1.000 |
|  | 51-60 years | -0.009 | 0.003 | *0.013* |
|  | 61-70 years | -0.012 | 0.003 | *0.000* |
|  | 71-80 years | -0.013 | 0.003 | *0.000* |
|  | 81-90 years | -0.029 | 0.004 | *0.000* |
| 41-50 years | 21-30 years | 0.003 | 0.003 | 1.000 |
|  | 31-40 years | 0.005 | 0.003 | 1.000 |
|  | 51-60 years | -0.003 | 0.002 | 1.000 |
|  | 61-70 years | -0.007 | 0.003 | 0.127 |
|  | 71-80 years | -0.007 | 0.003 | 0.117 |
|  | 81-90 years | -0.024 | 0.004 | *0.000* |
| 51-60 years | 21-30 years | 0.006 | 0.002 | *0.026* |
|  | 31-40 years | 0.009 | 0.003 | *0.013* |
|  | 41-50 years | 0.003 | 0.002 | 1.000 |
|  | 61-70 years | -0.004 | 0.001 | *0.043* |
|  | 71-80 years | -0.004 | 0.001 | 0.081 |
|  | 81-90 years | -0.020 | 0.003 | *0.000* |
| 61-70 years | 21-30 years | 0.010 | 0.002 | *0.000* |
|  | 31-40 years | 0.012 | 0.003 | *0.000* |
|  | 41-50 years | 0.007 | 0.003 | 0.127 |
|  | 51-60 years | 0.004 | 0.001 | *0.043* |
|  | 71-80 years | 0.000 | 0.001 | 1.000 |
|  | 81-90 years | -0.017 | 0.003 | *0.000* |
| 71-80 years | 21-30 years | 0.010 | 0.002 | *0.000* |
|  | 31-40 years | 0.013 | 0.003 | *0.000* |
|  | 41-50 years | 0.007 | 0.003 | 0.117 |
|  | 51-60 years | 0.004 | 0.001 | 0.081 |
|  | 61-70 years | 0.000 | 0.001 | 1.000 |
|  | 81-90 years | -0.016 | 0.003 | *0.000* |
| 81-90 years | 21-30 years | 0.027 | 0.004 | *0.000* |
|  | 31-40 years | 0.029 | 0.004 | *0.000* |
|  | 41-50 years | 0.024 | 0.004 | *0.000* |
|  | 51-60 years | 0.020 | 0.003 | *0.000* |
|  | 61-70 years | 0.017 | 0.003 | *0.000* |
|  | 71-80 years | 0.016 | 0.003 | *0.000* |
| Subregion 12 | Comparison | Mean difference | Standard error | P-value |
| 21-30 years | 31-40 years | 0.002 | 0.002 | 1.000 |
|  | 41-50 years | 0.006 | 0.002 | 0.279 |
|  | 51-60 years | 0.003 | 0.001 | 0.670 |
|  | 61-70 years | 0.006 | 0.001 | *0.005* |
|  | 71-80 years | 0.009 | 0.002 | *0.000* |
|  | 81-90 years | 0.019 | 0.003 | *0.000* |
| 31-40 years | 21-30 years | -0.002 | 0.002 | 1.000 |
|  | 41-50 years | 0.004 | 0.003 | 1.000 |
|  | 51-60 years | 0.001 | 0.002 | 1.000 |
|  | 61-70 years | 0.004 | 0.002 | 0.884 |
|  | 71-80 years | 0.007 | 0.002 | *0.006* |
|  | 81-90 years | 0.018 | 0.003 | *0.000* |
| 41-50 years | 21-30 years | -0.006 | 0.002 | 0.279 |
|  | 31-40 years | -0.004 | 0.003 | 1.000 |
|  | 51-60 years | -0.002 | 0.002 | 1.000 |
|  | 61-70 years | 0.000 | 0.002 | 1.000 |
|  | 71-80 years | 0.003 | 0.002 | 1.000 |
|  | 81-90 years | 0.014 | 0.003 | *0.000* |
| 51-60 years | 21-30 years | -0.003 | 0.001 | 0.670 |
|  | 31-40 years | -0.001 | 0.002 | 1.000 |
|  | 41-50 years | 0.002 | 0.002 | 1.000 |
|  | 61-70 years | 0.002 | 0.001 | 0.124 |
|  | 71-80 years | 0.006 | 0.001 | *0.000* |
|  | 81-90 years | 0.016 | 0.002 | *0.000* |
| 61-70 years | 21-30 years | -0.006 | 0.001 | *0.005* |
|  | 31-40 years | -0.004 | 0.002 | 0.884 |
|  | 41-50 years | 0.000 | 0.002 | 1.000 |
|  | 51-60 years | -0.002 | 0.001 | 0.124 |
|  | 71-80 years | 0.003 | 0.001 | *0.036* |
|  | 81-90 years | 0.014 | 0.002 | *0.000* |
| 71-80 years | 21-30 years | -0.009 | 0.002 | *0.000* |
|  | 31-40 years | -0.007 | 0.002 | *0.006* |
|  | 41-50 years | -0.003 | 0.002 | 1.000 |
|  | 51-60 years | -0.006 | 0.001 | *0.000* |
|  | 61-70 years | -0.003 | 0.001 | *0.036* |
|  | 81-90 years | 0.010 | 0.002 | *0.001* |
| 81-90 years | 21-30 years | -0.019 | 0.003 | *0.000* |
|  | 31-40 years | -0.018 | 0.003 | *0.000* |
|  | 41-50 years | -0.014 | 0.003 | *0.000* |
|  | 51-60 years | -0.016 | 0.002 | *0.000* |
|  | 61-70 years | -0.014 | 0.002 | *0.000* |
|  | 71-80 years | -0.010 | 0.002 | *0.001* |
| Subregion 13 | Comparison | Mean difference | Standard error | P-value |
| 21-30 years | 31-40 years | 0.000 | 0.003 | 1.000 |
|  | 41-50 years | 0.001 | 0.003 | 1.000 |
|  | 51-60 years | 0.000 | 0.002 | 1.000 |
|  | 61-70 years | 0.008 | 0.002 | *0.002* |
|  | 71-80 years | 0.021 | 0.002 | *0.000* |
|  | 81-90 years | 0.035 | 0.004 | *0.000* |
| 31-40 years | 21-30 years | 0.000 | 0.003 | 1.000 |
|  | 41-50 years | 0.000 | 0.003 | 1.000 |
|  | 51-60 years | 0.000 | 0.003 | 1.000 |
|  | 61-70 years | 0.008 | 0.003 | 0.061 |
|  | 71-80 years | 0.020 | 0.003 | *0.000* |
|  | 81-90 years | 0.034 | 0.004 | *0.000* |
| 41-50 years | 21-30 years | -0.001 | 0.003 | 1.000 |
|  | 31-40 years | 0.000 | 0.003 | 1.000 |
|  | 51-60 years | 0.000 | 0.002 | 1.000 |
|  | 61-70 years | 0.007 | 0.003 | 0.086 |
|  | 71-80 years | 0.020 | 0.003 | *0.000* |
|  | 81-90 years | 0.034 | 0.004 | *0.000* |
| 51-60 years | 21-30 years | 0.000 | 0.002 | 1.000 |
|  | 31-40 years | 0.000 | 0.003 | 1.000 |
|  | 41-50 years | 0.000 | 0.002 | 1.000 |
|  | 61-70 years | 0.007 | 0.001 | *0.000* |
|  | 71-80 years | 0.020 | 0.001 | *0.000* |
|  | 81-90 years | 0.034 | 0.003 | *0.000* |
| 61-70 years | 21-30 years | -0.008 | 0.002 | *0.002* |
|  | 31-40 years | -0.008 | 0.003 | 0.061 |
|  | 41-50 years | -0.007 | 0.003 | 0.086 |
|  | 51-60 years | -0.007 | 0.001 | *0.000* |
|  | 71-80 years | 0.013 | 0.001 | *0.000* |
|  | 81-90 years | 0.027 | 0.003 | *0.000* |
| 71-80 years | 21-30 years | -0.021 | 0.002 | *0.000* |
|  | 31-40 years | -0.020 | 0.003 | *0.000* |
|  | 41-50 years | -0.020 | 0.003 | *0.000* |
|  | 51-60 years | -0.020 | 0.001 | *0.000* |
|  | 61-70 years | -0.013 | 0.001 | *0.000* |
|  | 81-90 years | 0.014 | 0.003 | *0.001* |
| 81-90 years | 21-30 years | -0.035 | 0.004 | *0.000* |
|  | 31-40 years | -0.034 | 0.004 | *0.000* |
|  | 41-50 years | -0.034 | 0.004 | *0.000* |
|  | 51-60 years | -0.034 | 0.003 | *0.000* |
|  | 61-70 years | -0.027 | 0.003 | *0.000* |
|  | 71-80 years | -0.014 | 0.003 | *0.001* |
| Subregion 14 | Comparison | Mean difference | Standard error | P-value |
| 21-30 years | 31-40 years | 0.000 | 0.000 | 1.000 |
|  | 41-50 years | 0.000 | 0.000 | 1.000 |
|  | 51-60 years | 0.000 | 0.000 | 1.000 |
|  | 61-70 years | -0.001 | 0.000 | 1.000 |
|  | 71-80 years | -0.001 | 0.000 | 0.709 |
|  | 81-90 years | 0.000 | 0.001 | 1.000 |
| 31-40 years | 21-30 years | 0.000 | 0.000 | 1.000 |
|  | 41-50 years | 0.000 | 0.001 | 1.000 |
|  | 51-60 years | 0.000 | 0.000 | 1.000 |
|  | 61-70 years | 0.000 | 0.000 | 1.000 |
|  | 71-80 years | 0.000 | 0.000 | 1.000 |
|  | 81-90 years | 0.001 | 0.001 | 1.000 |
| 41-50 years | 21-30 years | 0.000 | 0.000 | 1.000 |
|  | 31-40 years | 0.000 | 0.001 | 1.000 |
|  | 51-60 years | -0.001 | 0.000 | 1.000 |
|  | 61-70 years | -0.001 | 0.000 | 1.000 |
|  | 71-80 years | -0.001 | 0.000 | 0.768 |
|  | 81-90 years | 0.000 | 0.001 | 1.000 |
| 51-60 years | 21-30 years | 0.000 | 0.000 | 1.000 |
|  | 31-40 years | 0.000 | 0.000 | 1.000 |
|  | 41-50 years | 0.001 | 0.000 | 1.000 |
|  | 61-70 years | 0.000 | 0.000 | 1.000 |
|  | 71-80 years | 0.000 | 0.000 | 1.000 |
|  | 81-90 years | 0.001 | 0.000 | 1.000 |
| 61-70 years | 21-30 years | 0.001 | 0.000 | 1.000 |
|  | 31-40 years | 0.000 | 0.000 | 1.000 |
|  | 41-50 years | 0.001 | 0.000 | 1.000 |
|  | 51-60 years | 0.000 | 0.000 | 1.000 |
|  | 71-80 years | 0.000 | 0.000 | 1.000 |
|  | 81-90 years | 0.001 | 0.000 | 0.974 |
| 71-80 years | 21-30 years | 0.001 | 0.000 | 0.709 |
|  | 31-40 years | 0.000 | 0.000 | 1.000 |
|  | 41-50 years | 0.001 | 0.000 | 0.768 |
|  | 51-60 years | 0.000 | 0.000 | 1.000 |
|  | 61-70 years | 0.000 | 0.000 | 1.000 |
|  | 81-90 years | 0.001 | 0.000 | 0.637 |
| 81-90 years | 21-30 years | 0.000 | 0.001 | 1.000 |
|  | 31-40 years | -0.001 | 0.001 | 1.000 |
|  | 41-50 years | 0.000 | 0.001 | 1.000 |
|  | 51-60 years | -0.001 | 0.000 | 1.000 |
|  | 61-70 years | -0.001 | 0.000 | 0.974 |
|  | 71-80 years | -0.001 | 0.000 | 0.637 |
| Subregion 15 | Comparison | Mean difference | Standard error | P-value |
| 21-30 years | 31-40 years | 0.001 | 0.001 | 1.000 |
|  | 41-50 years | 0.002 | 0.001 | 1.000 |
|  | 51-60 years | 0.000 | 0.001 | 1.000 |
|  | 61-70 years | -0.002 | 0.001 | 1.000 |
|  | 71-80 years | -0.003 | 0.001 | *0.019* |
|  | 81-90 years | -0.010 | 0.002 | *0.000* |
| 31-40 years | 21-30 years | -0.001 | 0.001 | 1.000 |
|  | 41-50 years | 0.001 | 0.002 | 1.000 |
|  | 51-60 years | -0.001 | 0.001 | 1.000 |
|  | 61-70 years | -0.003 | 0.001 | 0.490 |
|  | 71-80 years | -0.005 | 0.001 | *0.007* |
|  | 81-90 years | -0.011 | 0.002 | *0.000* |
| 41-50 years | 21-30 years | -0.002 | 0.001 | 1.000 |
|  | 31-40 years | -0.001 | 0.002 | 1.000 |
|  | 51-60 years | -0.002 | 0.001 | 0.939 |
|  | 61-70 years | -0.004 | 0.001 | *0.016* |
|  | 71-80 years | -0.006 | 0.001 | *0.000* |
|  | 81-90 years | -0.012 | 0.002 | *0.000* |
| 51-60 years | 21-30 years | 0.000 | 0.001 | 1.000 |
|  | 31-40 years | 0.001 | 0.001 | 1.000 |
|  | 41-50 years | 0.002 | 0.001 | 0.939 |
|  | 61-70 years | -0.002 | 0.001 | *0.048* |
|  | 71-80 years | -0.003 | 0.001 | *0.000* |
|  | 81-90 years | -0.010 | 0.002 | *0.000* |
| 61-70 years | 21-30 years | 0.002 | 0.001 | 1.000 |
|  | 31-40 years | 0.003 | 0.001 | 0.490 |
|  | 41-50 years | 0.004 | 0.001 | *0.016* |
|  | 51-60 years | 0.002 | 0.001 | *0.048* |
|  | 71-80 years | -0.002 | 0.001 | 0.196 |
|  | 81-90 years | -0.008 | 0.002 | *0.000* |
| 71-80 years | 21-30 years | 0.003 | 0.001 | *0.019* |
|  | 31-40 years | 0.005 | 0.001 | *0.007* |
|  | 41-50 years | 0.006 | 0.001 | *0.000* |
|  | 51-60 years | 0.003 | 0.001 | *0.000* |
|  | 61-70 years | 0.002 | 0.001 | 0.196 |
|  | 81-90 years | -0.006 | 0.002 | *0.001* |
| 81-90 years | 21-30 years | 0.010 | 0.002 | *0.000* |
|  | 31-40 years | 0.011 | 0.002 | *0.000* |
|  | 41-50 years | 0.012 | 0.002 | *0.000* |
|  | 51-60 years | 0.010 | 0.002 | *0.000* |
|  | 61-70 years | 0.008 | 0.002 | *0.000* |
|  | 71-80 years | 0.006 | 0.002 | *0.001* |
| Subregion 16 | Comparison | Mean difference | Standard error | P-value |
| 21-30 years | 31-40 years | -0.002 | 0.006 | 1.000 |
|  | 41-50 years | -0.003 | 0.006 | 1.000 |
|  | 51-60 years | 0.007 | 0.004 | 1.000 |
|  | 61-70 years | 0.003 | 0.004 | 1.000 |
|  | 71-80 years | 0.007 | 0.005 | 1.000 |
|  | 81-90 years | 0.022 | 0.008 | 0.079 |
| 31-40 years | 21-30 years | 0.002 | 0.006 | 1.000 |
|  | 41-50 years | -0.001 | 0.007 | 1.000 |
|  | 51-60 years | 0.009 | 0.005 | 1.000 |
|  | 61-70 years | 0.004 | 0.005 | 1.000 |
|  | 71-80 years | 0.009 | 0.006 | 1.000 |
|  | 81-90 years | 0.024 | 0.008 | 0.091 |
| 41-50 years | 21-30 years | 0.003 | 0.006 | 1.000 |
|  | 31-40 years | 0.001 | 0.007 | 1.000 |
|  | 51-60 years | 0.010 | 0.005 | 1.000 |
|  | 61-70 years | 0.006 | 0.005 | 1.000 |
|  | 71-80 years | 0.010 | 0.006 | 1.000 |
|  | 81-90 years | 0.025 | 0.008 | 0.057 |
| 51-60 years | 21-30 years | -0.007 | 0.004 | 1.000 |
|  | 31-40 years | -0.009 | 0.005 | 1.000 |
|  | 41-50 years | -0.010 | 0.005 | 1.000 |
|  | 61-70 years | -0.005 | 0.002 | 1.000 |
|  | 71-80 years | 0.000 | 0.003 | 1.000 |
|  | 81-90 years | 0.015 | 0.007 | 0.595 |
| 61-70 years | 21-30 years | -0.003 | 0.004 | 1.000 |
|  | 31-40 years | -0.004 | 0.005 | 1.000 |
|  | 41-50 years | -0.006 | 0.005 | 1.000 |
|  | 51-60 years | 0.005 | 0.002 | 1.000 |
|  | 71-80 years | 0.004 | 0.003 | 1.000 |
|  | 81-90 years | 0.020 | 0.007 | 0.099 |
| 71-80 years | 21-30 years | -0.007 | 0.005 | 1.000 |
|  | 31-40 years | -0.009 | 0.006 | 1.000 |
|  | 41-50 years | -0.010 | 0.006 | 1.000 |
|  | 51-60 years | 0.000 | 0.003 | 1.000 |
|  | 61-70 years | -0.004 | 0.003 | 1.000 |
|  | 81-90 years | 0.015 | 0.007 | 0.620 |
| 81-90 years | 21-30 years | -0.022 | 0.008 | 0.079 |
|  | 31-40 years | -0.024 | 0.008 | 0.091 |
|  | 41-50 years | -0.025 | 0.008 | 0.057 |
|  | 51-60 years | -0.015 | 0.007 | 0.595 |
|  | 61-70 years | -0.020 | 0.007 | 0.099 |
|  | 71-80 years | -0.015 | 0.007 | 0.620 |
| Subregion 17 | Comparison | Mean difference | Standard error | P-value |
| 21-30 years | 31-40 years | 0.001 | 0.003 | 1.000 |
|  | 41-50 years | -0.004 | 0.003 | 1.000 |
|  | 51-60 years | 0.000 | 0.002 | 1.000 |
|  | 61-70 years | 0.010 | 0.002 | *0.000* |
|  | 71-80 years | 0.023 | 0.002 | *0.000* |
|  | 81-90 years | 0.033 | 0.004 | *0.000* |
| 31-40 years | 21-30 years | -0.001 | 0.003 | 1.000 |
|  | 41-50 years | -0.005 | 0.004 | 1.000 |
|  | 51-60 years | 0.000 | 0.003 | 1.000 |
|  | 61-70 years | 0.009 | 0.003 | *0.025* |
|  | 71-80 years | 0.022 | 0.003 | *0.000* |
|  | 81-90 years | 0.032 | 0.004 | *0.000* |
| 41-50 years | 21-30 years | 0.004 | 0.003 | 1.000 |
|  | 31-40 years | 0.005 | 0.004 | 1.000 |
|  | 51-60 years | 0.004 | 0.003 | 1.000 |
|  | 61-70 years | 0.014 | 0.003 | *0.000* |
|  | 71-80 years | 0.027 | 0.003 | *0.000* |
|  | 81-90 years | 0.037 | 0.004 | *0.000* |
| 51-60 years | 21-30 years | 0.000 | 0.002 | 1.000 |
|  | 31-40 years | 0.000 | 0.003 | 1.000 |
|  | 41-50 years | -0.004 | 0.003 | 1.000 |
|  | 61-70 years | 0.010 | 0.001 | *0.000* |
|  | 71-80 years | 0.022 | 0.001 | *0.000* |
|  | 81-90 years | 0.033 | 0.004 | *0.000* |
| 61-70 years | 21-30 years | -0.010 | 0.002 | *0.000* |
|  | 31-40 years | -0.009 | 0.003 | *0.025* |
|  | 41-50 years | -0.014 | 0.003 | *0.000* |
|  | 51-60 years | -0.010 | 0.001 | *0.000* |
|  | 71-80 years | 0.013 | 0.002 | *0.000* |
|  | 81-90 years | 0.023 | 0.004 | *0.000* |
| 71-80 years | 21-30 years | -0.023 | 0.002 | *0.000* |
|  | 31-40 years | -0.022 | 0.003 | *0.000* |
|  | 41-50 years | -0.027 | 0.003 | *0.000* |
|  | 51-60 years | -0.022 | 0.001 | *0.000* |
|  | 61-70 years | -0.013 | 0.002 | *0.000* |
|  | 81-90 years | 0.010 | 0.004 | 0.097 |
| 81-90 years | 21-30 years | -0.033 | 0.004 | *0.000* |
|  | 31-40 years | -0.032 | 0.004 | *0.000* |
|  | 41-50 years | -0.037 | 0.004 | *0.000* |
|  | 51-60 years | -0.033 | 0.004 | *0.000* |
|  | 61-70 years | -0.023 | 0.004 | *0.000* |
|  | 71-80 years | -0.010 | 0.004 | 0.097 |
